# Muscle weakness precedes atrophy during cancer cachexia and is linked to muscle-specific mitochondrial stress

**DOI:** 10.1172/jci.insight.155147

**Published:** 2022-12-22

**Authors:** Luca J. Delfinis, Catherine A. Bellissimo, Shivam Gandhi, Sara N. DiBenedetto, Madison C. Garibotti, Arshdeep K. Thuhan, Stavroula Tsitkanou, Megan E. Rosa-Caldwell, Fasih A. Rahman, Arthur J. Cheng, Michael P. Wiggs, Uwe Schlattner, Joe Quadrilatero, Nicholas P. Greene, Christopher G.R. Perry

**Affiliations:** 1 Muscle Health Research Centre, School of Kinesiology, Faculty of Health, York University, Toronto, Ontario, Canada.; 2 Cachexia Research Laboratory, Department of Health, Human Performance and Recreation, College of Education and Health Professions, University of Arkansas, Fayetteville, Arkansas, USA.; 3 Department of Kinesiology and Health Sciences, Faculty of Health, University of Waterloo, Waterloo, Ontario, Canada.; 4 Mooney Lab for Exercise, Nutrition, and Biochemistry, Department of Health, Human Performance, and Recreation, Baylor University, Waco, Texas, USA.; 5 Laboratory of Fundamental and Applied Bioenergetics, University Grenoble Alpes and INSERM U1055, Grenoble, France, and Institut Universitaire de France, Paris, France.

**Keywords:** Metabolism, Oncology, Colorectal cancer, Mitochondria, Skeletal muscle

## Abstract

Muscle weakness and wasting are defining features of cancer-induced cachexia. Mitochondrial stress occurs before atrophy in certain muscles, but the possibility of heterogeneous responses between muscles and across time remains unclear. Using mice inoculated with Colon-26 cancer, we demonstrate that specific force production was reduced in quadriceps and diaphragm at 2 weeks in the absence of atrophy. At this time, pyruvate-supported mitochondrial respiration was lower in quadriceps while mitochondrial H_2_O_2_ emission was elevated in diaphragm. By 4 weeks, atrophy occurred in both muscles, but specific force production increased to control levels in quadriceps such that reductions in absolute force were due entirely to atrophy. Specific force production remained reduced in diaphragm. Mitochondrial respiration increased and H_2_O_2_ emission was unchanged in both muscles versus control while mitochondrial creatine sensitivity was reduced in quadriceps. These findings indicate muscle weakness precedes atrophy and is linked to heterogeneous mitochondrial alterations that could involve adaptive responses to metabolic stress. Eventual muscle-specific restorations in specific force and bioenergetics highlight how the effects of cancer on one muscle do not predict the response in another muscle. Exploring heterogeneous responses of muscle to cancer may reveal new mechanisms underlying distinct sensitivities, or resistance, to cancer cachexia.

## Introduction

Cancer-induced cachexia is a multifactorial syndrome characterized, in part, by a loss of skeletal muscle mass that cannot be fully reversed by conventional nutritional support (1). This condition leads to progressive reductions in functional independence and quality of life (2). Such declines in muscle mass also reduce tolerance to anticancer therapies and overall survivability (3, 4) and are associated with increased hospitalization time (5). It is thought that 20%–80% of cancer patients develop cachexia depending on the type and stage of cancer (6). However, the time-dependent relationship between muscle atrophy and weakness remains unclear, as does the degree to which this relationship may vary between muscle types. Exploring the natural divergence of muscle responses to cancer may be an opportunistic approach to identify distinct mechanisms underlying muscle weakness and wasting during cancer cachexia.

Contemporary hypotheses posit that muscle wasting during cachexia is induced by circulating factors generated during cancer, which trigger protein degradation and loss of myofibrillar proteins through various mechanisms (4, 7, 8). However, recent literature suggests skeletal muscle mitochondria are also subject to damage during cancer cachexia (9–11) and may be direct contributors to either muscle weakness or atrophy. Current literature suggests oxidative phosphorylation is impaired in the soleus, gastrocnemius, and plantaris muscle of tumor-bearing mice, while reactive oxygen species (ROS) — in the form of mitochondrial H_2_O_2_ emission (mH_2_O_2_) — can be increased or decreased depending on the muscle and duration of cancer (9, 11, 12). This suggests cellular mechanisms contributing to muscle loss during cancer cachexia may be more complicated than previously believed. Moreover, in the Lewis lung carcinoma (LLC) xenograft mouse model, certain indices of skeletal muscle mitochondrial dysfunction preceded the onset of muscle atrophy, suggesting mitochondria may be a potential therapeutic target (12). This concept was supported by subsequent studies reporting positive effects of the mitochondria-targeting compound SS-31 in preventing certain indices of cachexia in some but not all muscles of the Colon-26 (C26) xenograft mouse model (13, 14). However, given the multifactorial contributions to cachexia during cancer, it seems likely the relationship between mitochondria and myopathy may differ between muscle type and throughout cancer progression.

Indeed, skeletal muscle mitochondria are known to be highly adaptable to metabolic stressors and can super-compensate during an energy crisis (15, 16). In this light, the available literature does not provide sufficient information to predict the extent to which cancer will affect individual muscles, particularly in relation to their underlying mitochondrial responses to the systemic stress of this disease. Understanding the time-dependent nature of unique mitochondrial signatures during cancer-induced cachexia might better inform the development of mitochondrial therapies that have so far yielded disparate results across various muscle types in the C26 cancer mouse model (13, 14).

In this study, we compared the time-dependent relationship of muscle dysfunction and mitochondrial bioenergetic responses to cancer between locomotor (quadriceps) and respiratory muscles (diaphragm). In so doing, we employed a careful consideration of mitochondrial substrate titration protocols modeling key parameters governing mitochondrial bioenergetics in vivo. Similar assay design considerations have been essential for identifying precise mitochondrial bioenergetic contributions to cellular function in our previous research (17–20). Using the C26 tumor–bearing mouse model, we reveal muscle weakness precedes atrophy in quadriceps and diaphragm. Energetic insufficiencies were more pronounced in quadriceps whereas mitochondrial redox stress was more evident in diaphragm, yet both muscles showed a delayed correction, if not super-compensation, as cancer progressed. In quadriceps, increased mitochondrial respiratory control was related to a surprising increase in specific force production at 4 weeks that likely mitigated the magnitude of reduction in absolute force due to atrophy. These findings demonstrate the effects of cancer on one muscle do not necessarily predict the response in another muscle type. Moreover, the heterogeneous muscle-specific and time-dependent mitochondrial relationships to cancer may provide an opportunity for informing a more targeted approach to developing mitochondrial therapies to improve muscle health in this debilitating disorder.

## Results

### C26 tumor–bearing mice show progressive reductions in body weight, muscle mass, fat mass, and grip strength.

**Figure 1:**
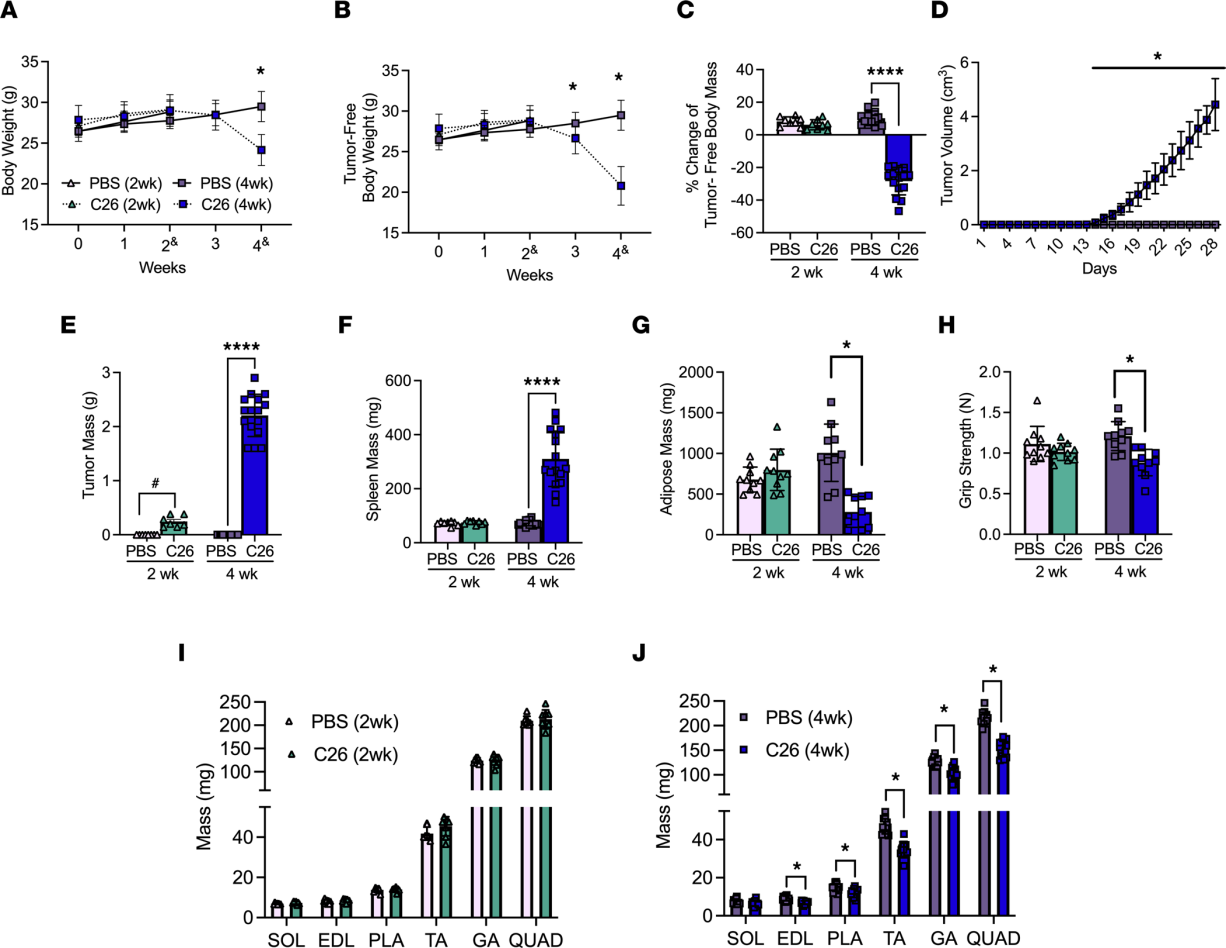
The effects of C26 colon cancer cells’ implantation on body size, tumor size, muscle mass’ and force. Analysis of CD2F1 mice with subcutaneous C26 implantations or with PBS were performed. Body weights (**A**, *n* = 8–16) and tumor-free body weights (**B**, *n* = 8–16) were analyzed every week (2^&^ mice were measured at a 14- to 17-day window, and 4^&^ mice were measured on a 26- to 29-day window). Percentage change in tumor-free body weights were analyzed from day 0 to endpoint (**C**, *n* = 8–16). In vivo tumor volume measurements were made using calipers (**D**, *n* = 16). Tumor mass (**E**, *n* = 7–16) and spleen mass (**F**, *n* = 8–16) measurements were also completed. Subcutaneous fat from the inguinal fat depot was weighed (**G**, *n* = 10). Grip strength was assessed in all groups (**H**, *n* = 10). Evaluations of hind limb muscle wet weights were made in the 2-week cohort (**I**, *n* = 8) and 4-week cohort (**J**, *n* = 16). Results represent mean ± SD; 2-tailed *t* tests were used to determine the difference between PBS(2wk) vs. C26(2wk) and PBS (4wk) vs. C26(4wk). One-way ANOVA was used to determine the difference between PBS(4wk) vs. C26(4wk) tumor growth. ^#^*P* < 0.05 PBS(2wk) vs. C26(2wk); *P < 0.05 PBS(4wk) vs. C26(4wk); ****P < 0.0001 PBS(4wk) vs. C26(4wk).

All results were in male mice. Body weights were reduced 4 weeks after subcutaneous implantations of C26 cells (Figure 1A), while tumor-free body weights progressively decreased beginning at 3 weeks to a net loss of 27% by 4 weeks (Figure 1, B and C) at a time of substantial tumor growth (Figure 1D). Tumors grew to approximately 0.2 g at 2 weeks and approximately 2.2 g at 4 weeks (Figure 1E). C26 spleen mass (marker of inflammatory stress) was not different from PBS at 2 weeks but was significantly greater at 4 weeks (Figure 1F). Adipose tissue from the inguinal fat pad was not different at 2 weeks but significantly lower at 4 weeks (–65%; Figure 1G). A similar pattern was observed with grip strength (Figure 1H). The mass of specific muscles was similar between C26 and PBS at 2 weeks (Figure 1I). At 4 weeks soleus (SOL) mass was similar between C26 and PBS while lower muscle masses were observed in C26 for extensor digitorum longus (EDL; –23%), plantaris (PLA; –20%), tibialis anterior (TA; –26%), gastrocnemius (GA; –21%), and quadriceps (QUAD; –29%) versus PBS (Figure 1J).

### Muscle atrophy occurs in both the quadriceps and diaphragm after 4 weeks of tumor bearing.

**Figure 2:**
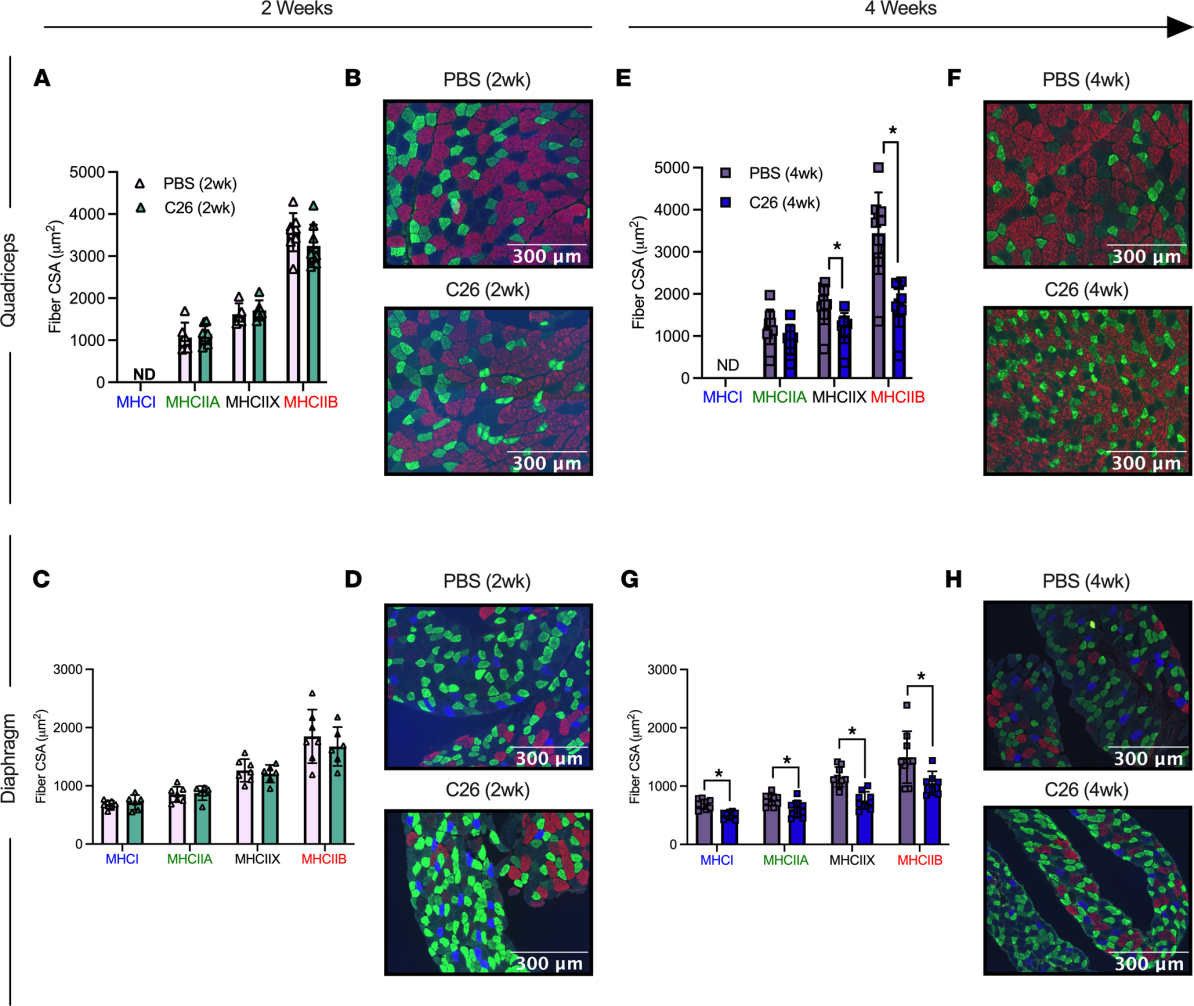
Evaluation of quadriceps and diaphragm fiber type atrophy in skeletal muscle from C26 tumor–bearing mice. Analysis of fiber histology on MHC isoforms of PBS and C26 mice was performed. CSA of MHC stains was evaluated in the quadriceps (**A**, *n* = 8; **B**, representative image; original magnification, ×20) and diaphragm at 2 weeks of tumor bearing (**C**, *n* = 6; **D**, representative image; original magnification, ×20). The same was completed for the quadriceps (**E**, *n* = 9; **F**, representative image; original magnification, ×20) and diaphragm at 4 weeks of tumor bearing (**G**, *n* = 9; **H**, representative image; original magnification, ×20). Results represent mean ± SD; 2-tailed *t* tests were used to determine the difference between PBS(2wk) versus C26(2wk) and PBS (4wk) versus C26(4wk). *P < 0.05 PBS (4wk) versus C26(4wk).

Muscle atrophy is a hallmark of cancer cachexia. In both quadriceps and diaphragm, fiber cross-sectional area (CSA) was similar between C26 and PBS groups for specific MHC isoforms (Figure 2, A–D) and when pooling all MHC isoforms (data not shown) at 2 weeks. However, at 4 weeks, quadriceps muscle exhibited lower CSA in pooled fibers (–40%, *P* < 0.05, data not shown) with specific reductions in MHC IIX (–32%) and MHC IIB (–49%) but not the MHC IIA isoform (Figure 2, E and F) versus PBS. MHC I–positive fibers were not detected in the quadriceps (Figure 2, B and F). At 4 weeks, diaphragm muscle also showed lower CSA in pooled fibers (–31%, *P* < 0.05, data not shown), which mirrored changes in MHC I (–28%), MHC IIA (–21%), MHC IIB (–30%), and MHC IIX (–35%) versus PBS at 4 weeks (Figure 2, G and H).

**Figure 3:**
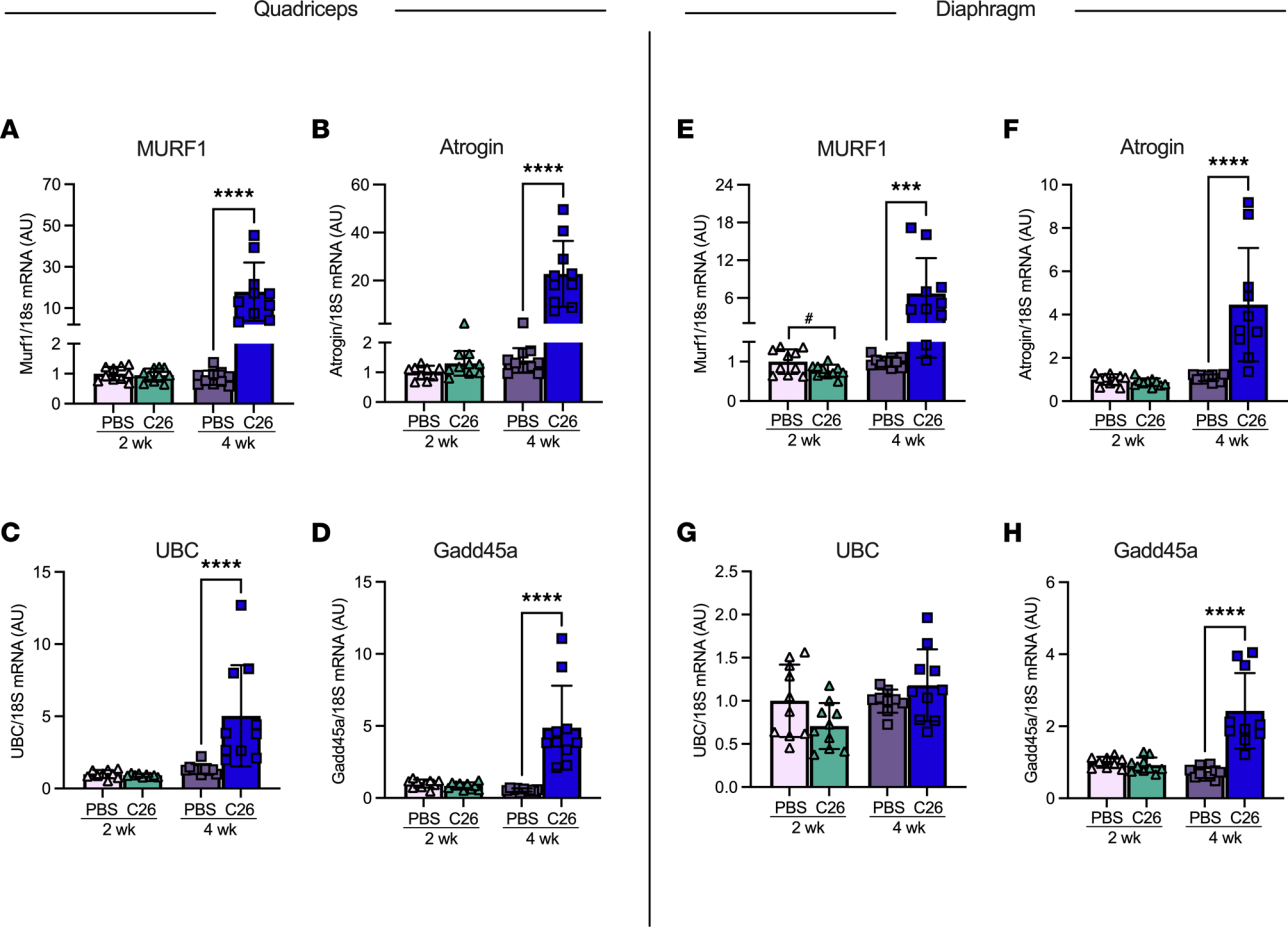
The effects of C26 colon cancer on the activation of atrophy markers in quadriceps and diaphragm muscle. mRNA content of atrophy markers muscle RING-finger protein-1 (MURF1) (**A**, *n* = 10), atrogin (**B**, *n* = 10), ubiquitin C (UBC) (**C**, *n* = 10), and growth arrest and DNA damage-inducible 45α (Gdd45a) (**D**, *n* = 10) was measured using quantitative PCR in the quadriceps of all groups. This was also completed in the diaphragm (**E**–**H**; *n* = 10). Results represent mean ± SD; 2-tailed *t* tests were used to determine the difference between PBS(2wk) versus C26(2wk) and PBS (4wk) versus C26(4wk). ^#^*P* < 0.05 PBS(2wk) versus C26(2wk); ***P < 0.001 PBS(4wk) versus C26(4wk); ****P < 0.0001 PBS(4wk) versus C26(4wk).

We validated that these reductions in fiber CSA were a result of muscle atrophy by measuring key proteasomal markers of muscle wasting. In the quadriceps, muscle RING-finger protein-1 (MURF1) mRNA content was significantly increased at 4 weeks of tumor bearing compared with PBS controls (Figure 3A). This was also the case for atrogin (Figure 3B), ubiquitin C (UBC; Figure 3C), and growth arrest and DNA damage-inducible 45α (Gadd45a; Figure 3D). The diaphragm demonstrated similar results, such that MURF1, atrogin, and Gadd45a were all significantly increased at 4 weeks of tumor bearing. However, this did not occur in UBC (Figure 3, E–H).

### Force production is reduced prior to atrophy in quadriceps and diaphragm but returns to normal in quadriceps.

**Figure 4:**
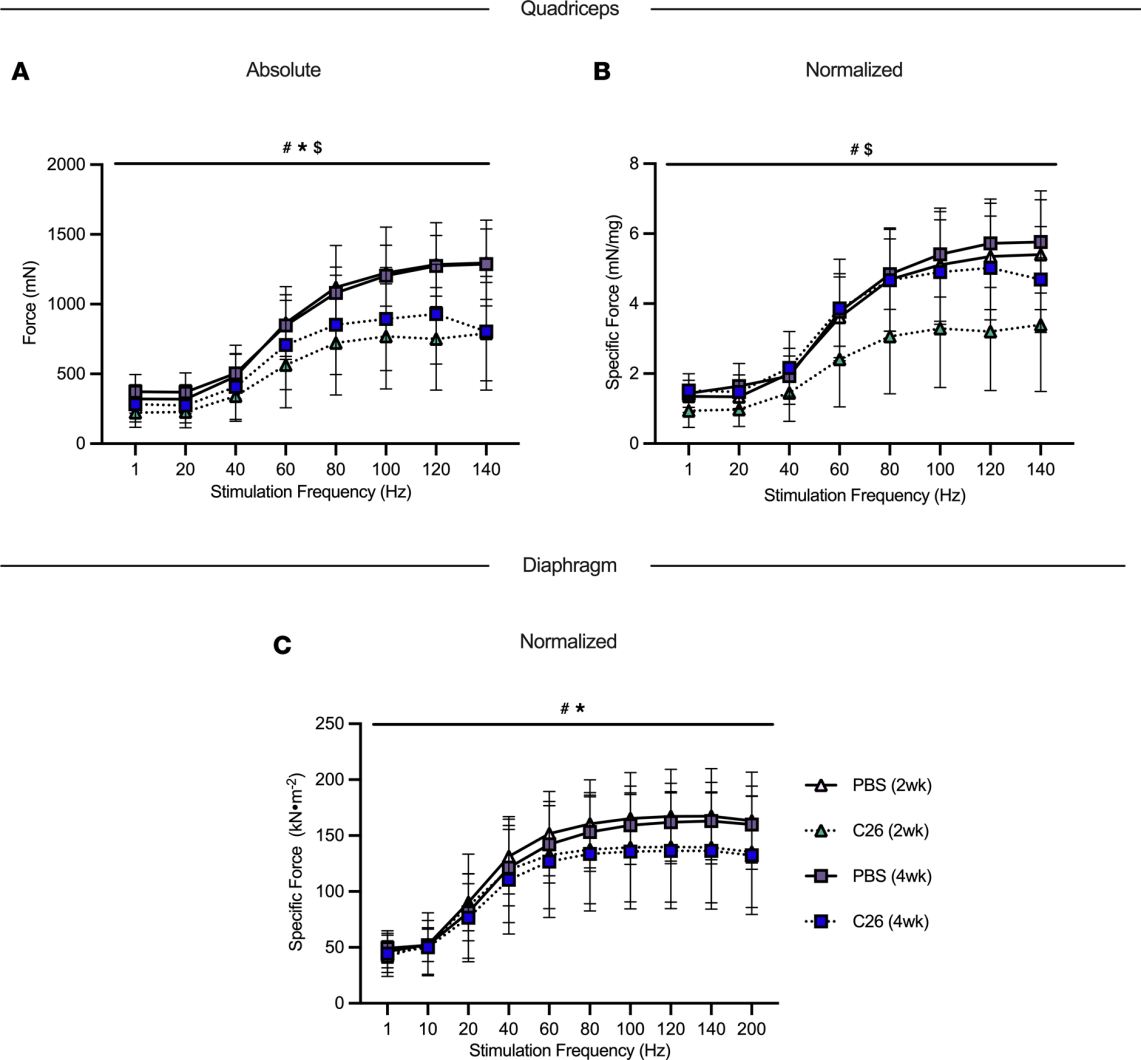
The effects of C26 colon cancer on quadriceps and diaphragm force production. In situ quadriceps force production was assessed using the force-frequency relationship (**A**, absolute force; **B**, normalized force to total quadriceps weight; *n* = 6–14) and in vitro diaphragm force production was also measured using the force-frequency relationship (**C**, normalized force; absolute force is not included as the method was performed on muscle strips; *n* = 6–12). Results represent mean ± SD; a 2-way ANOVA was used to determine the difference between PBS(2wk) versus C26(2wk) versus PBS (4wk) versus C26(4wk). ^#^*P* < 0.05 PBS(2wk) versus C26(2wk); *P < 0.05 PBS(4wk) versus C26(4wk); ^$^*P* < 0.05 C26 (2wk) versus C26 (4wk).

At 2 weeks and 4 weeks, C26 absolute muscle force was lower in quadriceps relative to PBS as a group main effect (Figure 4A). Interestingly, when absolute quadriceps force was normalized to muscle wet weight, specific force production remained decreased at 2 weeks of tumor bearing compared with PBS as a group main effect. However, at 4 weeks of tumor bearing, specific force was higher compared with 2 weeks and not different compared to PBS controls as a group main effect (Figure 4B). In addition, there was an interaction whereby C26 at 2 weeks produced less force at 80 Hz, 100 Hz, and 120 Hz compared with both PBS groups and the C26 group at 4 weeks (not shown).

We are unable to report absolute force in the diaphragm as dissected muscle strips of diaphragm were used to complete the in vitro force assay, not the whole diaphragm. At 2 weeks and 4 weeks of C26 tumor bearing, specific muscle force was lower in the diaphragm relative to PBS controls as a group main effect (Figure 4C).

### Mitochondrial electron transport chain protein contents are reduced in quadriceps but do not change in diaphragm by 4 weeks of tumor development.

**Figure 5:**
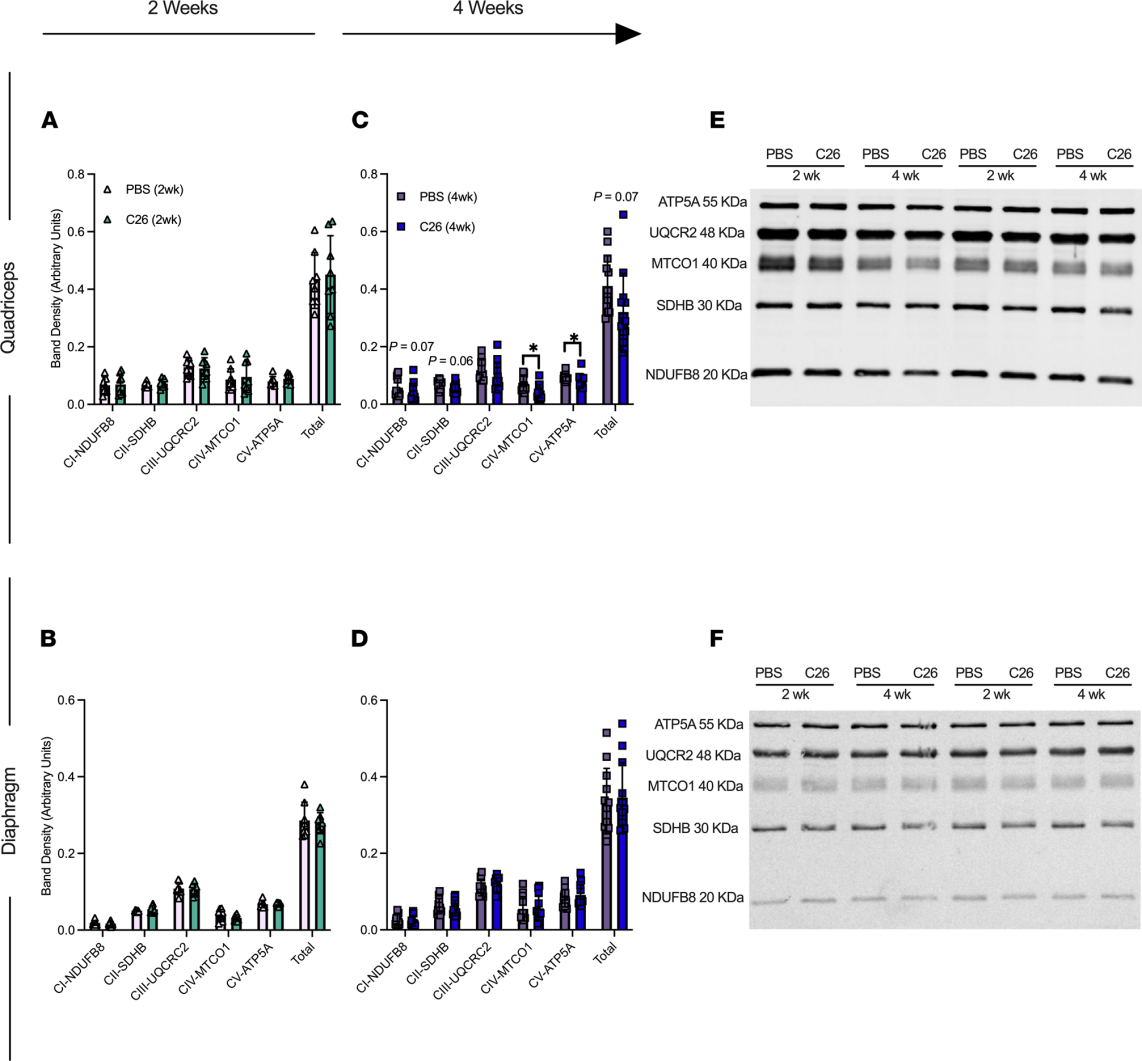
Muscle-specific changes in markers of ETC complexes in C26 tumor–bearing skeletal muscle. Protein content of ETC subunits was quantified in the quadriceps (**A**, *n* = 8) and diaphragm at 2 weeks (**B**, *n* = 8) and 4 weeks (**C** and **D**) (*n* = 12). (**E**) Representative blots for quadriceps and (**F**) representative blotsfor diaphragm. Results represent mean ± SD; 2-tailed *t* tests were used to determine the difference between PBS(2wk) versus C26(2wk) and PBS (4wk) versus C26(4wk). *P < 0.05 PBS (4wk) versus C26 (4wk).

At 2 weeks, electron transport chain (ETC) subunit contents in both muscles were unchanged in C26 relative to PBS controls (Figure 5, A and B). At 4 weeks, C26 showed lower contents in subunits of complex I (–31%), complex II (–18%), complex IV (–37%), complex V (–11%), and total ETC subunit content (–22%; Figure 5C) relative to PBS that were significant. ETC subunit contents did not change in diaphragm at 4 weeks relative to PBS (Figure 5D; representative blots, Figure 5, E and F).

### Mitochondrial respiratory control by ADP is greater in both muscles by 4 weeks of tumor development despite early reductions in the quadriceps.

**Figure 6:**
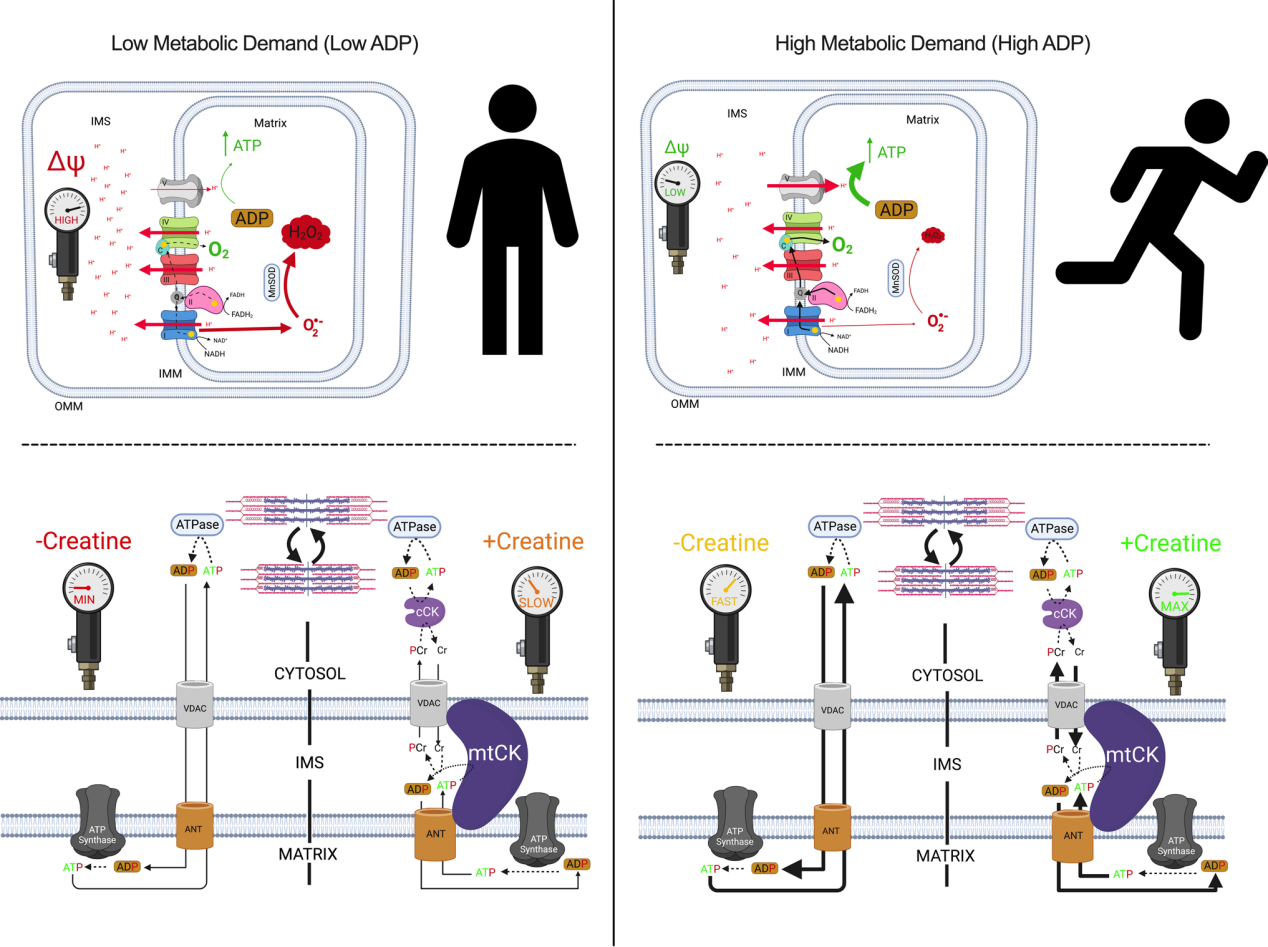
Schematic representation of energy homeostasis in states of low metabolic (left) versus high metabolic (right) demand. When ADP is low, less ATP is produced. A concomitant accumulation of [H^+^] in the inner membrane space (IMS) increases membrane potential (ΔΨ), attenuates [H^+^] pumping, induces premature electron slip, and generates superoxide (O_2_^•–^), which is dismutated to H_2_O_2_ by manganese superoxide dismutase (MnSOD; top left). Only complex I–derived O_2_^•–^ is displayed. When ADP is high, more ATP is produced as [H^+^] diffuses from the IMS to the mitochondrial matrix through ATP synthase. The decrease in ΔΨ lowers premature electron slip, generating less O_2_^•–^ and H_2_O_2_ (top right). ADP generated by ATPases throughout the cell enter the matrix through the voltage dependent anion channel (VDAC) on the outer mitochondrial membrane (OMM) and the adenine nucleotide translocase (ANT) on the inner mitochondrial membrane (IMM; bottom left). Creatine accelerates matrix ADP/ATP cycling and ATP synthesis by reducing the diffusion distance of the slower diffusing ADP and ATP while shuttling phosphate to the cytoplasm through rapidly diffusing phosphocreatine (PCr), which is used by cytosolic creatine kinase (cCK) to recycle local ATP to support the activity of various ATPases. Rapidly diffusing creatine returns to the IMS to be rephosphorylated by mitochondrial creatine kinase (mtCK). Non-ATPase sites of ATP hydrolysis are not displayed but also contribute to net metabolic demand (kinases and other ATP-dependent processes). The net effect of metabolic demand (global ATP hydrolysis) on matrix ADP/ATP cycling is displayed under the context of creatine-independent (-creatine) and creatine-dependent (+creatine) conditions. Figure adapted from Aliev et al., 2011; Guzun et al., 2012; Wallimann et al., 2011; and Nicholls and Ferguson, 2013 (23, 50–52). Created with BioRender.com.

We determined if the central role of ADP in stimulating respiration was impaired in both quadriceps and diaphragm at 2 and 4 weeks after subcutaneous implantations of C26 cancer cells. We stimulated complex I with NADH generated by pyruvate (5 mM) and malate (2 mM) across a range of ADP concentrations to challenge mitochondria with a spectrum of metabolic demands. The ADP titrations were repeated with (+creatine) and without (-creatine) 20 mM creatine to model the 2 main theoretical mechanisms of energy transfer from mitochondria to cytosolic compartments that utilize or bypass mitochondrial creatine kinase (mtCK), respectively (Figure 6). Briefly, the +creatine system stimulates mitochondria to export PCr whereas the -creatine condition drives ATP export.

**Figure 7:**
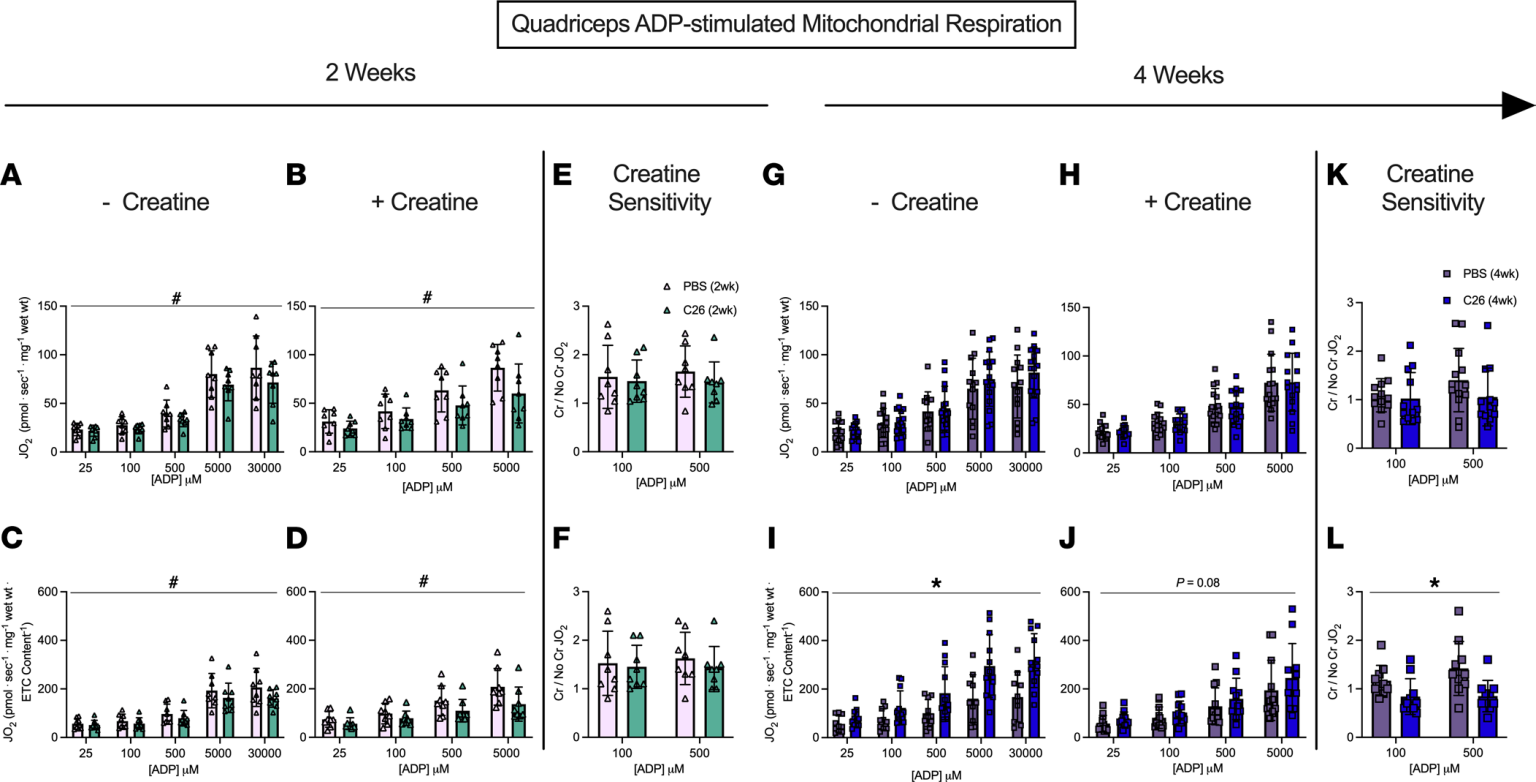
Complex I–supported mitochondrial respiration in quadriceps muscle of C26 tumor–bearing mice. ADP-stimulated (state III) respiration, supported by complex I–supported (NADH) substrates pyruvate (5 mM) and malate (2 mM), was assessed in the absence (-creatine) and presence (+creatine) of 20 mM creatine at a range of [ADP] until maximal respiration was achieved to model a spectrum of metabolic demands. Respiration was assessed in the quadriceps normalized to bundle size at 2 weeks (**A** and **B**) and normalized to ETC subunit content (**C** and **D**) to permit comparisons of intrinsic mitochondrial respiratory responses in each group. Creatine sensitivity was assessed by calculating the +creatine/-creatine ratio (**E** and **F**) given creatine normally increases ADP-stimulated respiration. The same measurements were completed at 4 weeks (**G**–**L**). Results represent mean ± SD; *n* = 8–16; a 2-way ANOVA was used to determine the difference between PBS(2wk) versus C26(2wk) and PBS (4wk) versus C26(4wk). ^#^*P* < 0.05, PBS(2wk) versus C26(2wk); *P < 0.05, PBS(4wk) versus C26(4wk).

In both the -creatine and +creatine conditions, pyruvate/malate-supported, ADP-stimulated respiration, normalized per mass of fiber bundles (not corrected for ETC subunit content), was lower 2 weeks after C26 implantations in the quadriceps compared with PBS, with a main effect across all ADP concentrations with or without creatine (Figure 7, A and B). The general reduction in respiration for C26 normalized per mass of fiber bundle was also seen when data were normalized to ETC subunit content (Figure 7, C and D). This suggests respiratory control was reduced within mitochondria due to an inherent property of the ETC not related to ETC abundance. We also evaluated if creatine sensitivity was altered in the C26 tumor–bearing muscle by calculating the +creatine/-creatine respiratory ratio. This creatine sensitivity index is a measure of the ability of creatine to stimulate respiration by accelerating matrix ADP/ATP cycling and represents coupling of the cCK system to ATP generation (Figure 6), particularly at submaximal ADP concentrations (21, 22), which we have reported previously (17, 18). In quadriceps, creatine sensitivities at 100 μM and 500 μM ADP were unchanged at 2 weeks in C26 versus PBS at this time point (Figure 7, E and F). Collectively, these findings indicate respiration was reduced to similar extents in both -creatine and +creatine conditions of energy exchange between mitochondria and cytoplasmic compartments.

These early decrements in quadriceps respiration in C26 at 2 weeks were reversed by 4 weeks. This apparent compensation was seen in both -creatine and +creatine conditions. Specifically, respiration was similar to PBS control mice at 4 weeks when normalized per mass of fiber bundle (Figure 7, G and H) and higher than controls when normalized to ETC subunit protein content (Figure 7, I and J) despite reductions in ETC content as described above (Figure 5C). These findings suggest mitochondria in quadriceps are highly plastic and can super-compensate by upregulating their responsiveness to ADP to levels exceeding PBS controls. Additionally, at 4 weeks, quadriceps mitochondrial creatine sensitivity was unchanged in C26 relative to PBS when respiration was normalized to bundle weight (Figure 7K). However, mitochondrial creatine sensitivity was impaired in C26 relative to PBS when considering respiration normalized to ETC subunit content given the ratio did not exceed a value of 1.0, which indicates that creatine could not stimulate respiration above the level elicited by ADP alone (Figure 7L). Thus, while C26 cancer strongly increased ADP-stimulated respiration by 4 weeks (Figure 7I), it compromised the coupling of creatine kinase energy transfer, suggesting that this system did not contribute to restored force at this time point (Figure 4B).

**Figure 8:**
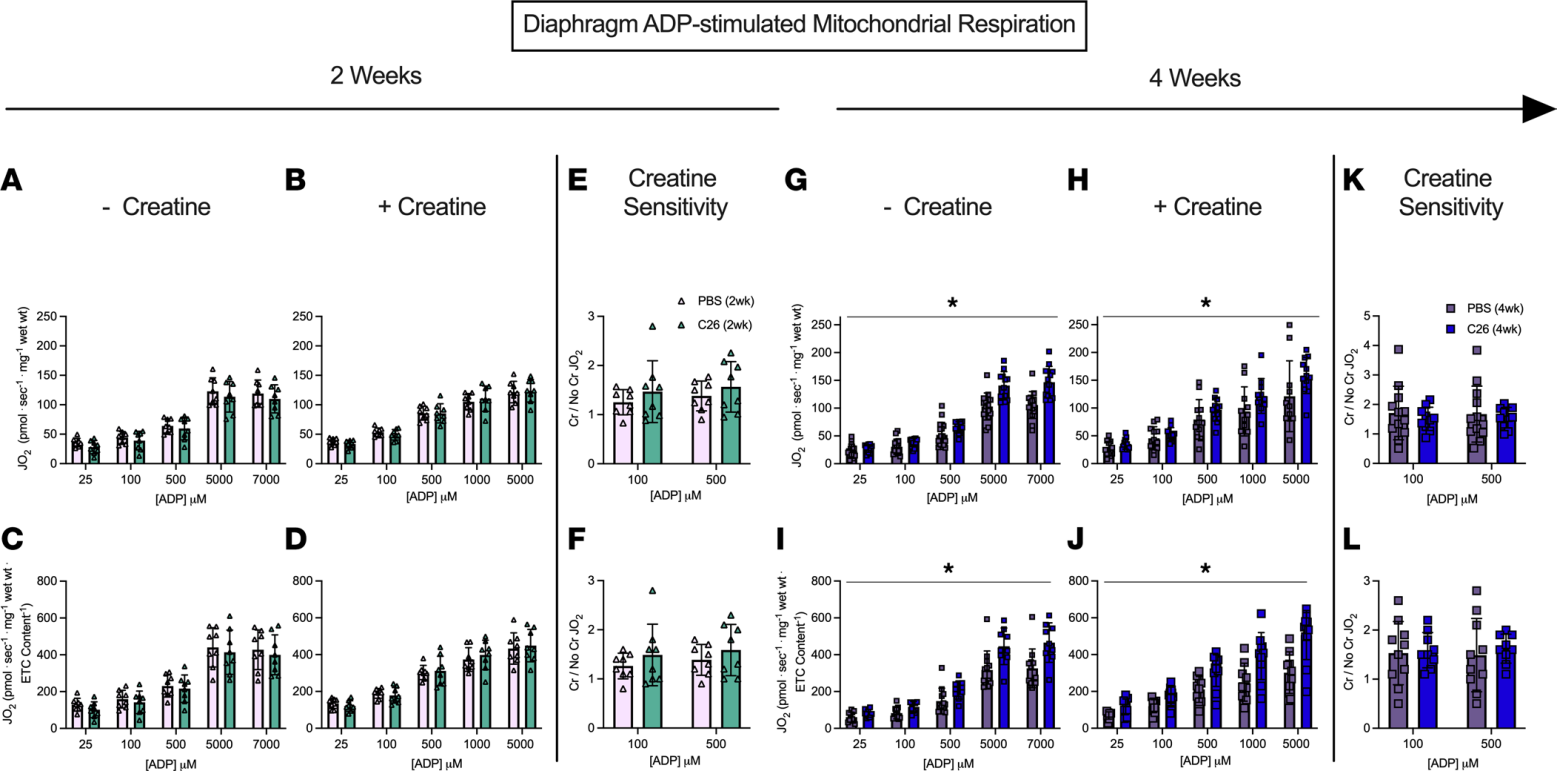
Complex I–supported mitochondrial respiration in diaphragm muscle of C26 tumor–bearing mice. ADP-stimulated (State III) respiration, supported by complex I–supported (NADH) substrates pyruvate (5 mM) and malate (2 mM), was assessed in the absence (-creatine) and presence (+creatine) of 20 mM creatine at a range of [ADP] until maximal respiration was achieved to model a spectrum of metabolic demands. Respiration was assessed in the diaphragm normalized to bundle size at 2 weeks (**A** and **B**) and normalized to ETC subunit content (**C** and **D**) to permit comparisons of intrinsic mitochondrial respiratory responses in each group. Creatine sensitivity was assessed by calculating the +creatine/-creatine ratio (**E** and **F**) given creatine normally increases ADP-stimulated respiration. The same measurements were completed at 4 weeks (**G**–**L**). Results represent mean ± SD; *n* = 8–16; a 2-way ANOVA was used to determine the difference between PBS(2wk) versus C26(2wk) and PBS (4wk) versus C26(4wk). *P < 0.05, PBS(4wk) versus C26(4wk).

In the diaphragm, respiration was similar between C26 and PBS at 2 weeks (Figure 8, A–F) but was greater in C26 compared with PBS at 4 weeks in both the -creatine and +creatine conditions (Figure 8, G–J). This upregulation by 4 weeks occurred despite no changes in ETC subunit content as noted above (Figure 5D), which suggests mitochondria increase their responsiveness to ADP through mechanisms that may be independent of mitochondrial content. No changes in creatine sensitivity were observed in C26 versus PBS at 4 weeks (Figure 8, K and L), suggesting that coupling of creatine kinase to ATP generation was maintained, in contrast to impaired creatine sensitivity seen in the quadriceps as noted above. Last, there was a significant interaction whereby respiration was greater in C26 versus PBS at 5,000 μM and 7,000 μM ADP when normalized per mass of fiber bundles (Figure 8, G and H) and at all ADP concentrations except 25 μM and 100 μM when normalized to total ETC subunit content (Figure 8, I and J).

These alterations were specific to pyruvate/malate-supported ADP-stimulated respiration as there were no changes in respiration in response to glutamate (further NADH generation) and succinate (FADH_2_) generation when comparing C26 to PBS at either time point (Supplemental Figures 1 and 2; supplemental material available online with this article; https://doi.org/10.1172/jci.insight.155147DS1). By 2 weeks of C26, diaphragm showed a decrease in state II respiration (no ADP, supported by pyruvate/malate; Supplemental Figures 1 and 2), which is generally used as a marker of respiration driven by proton leak into the matrix from the inner membrane space through various sites that are not coupled to ATP synthesis (23). However, state II respiration was greater than control by 4 weeks in both muscles, suggesting greater uncoupling that occurs as cancer progresses (Supplemental Figure 2). Last, changes in respiration noted above did not result in changes to the phosphorylation of AMPK in C26 relative to PBS at either 2- or 4-week time points (Supplemental Figure 3). However, increases in AMPK and the phosphorylated to total AMPK (p-AMPK/AMPK) ratio were trending in the C26 (4wk) group in the quadriceps.

### H_2_O_2_ emission is increased in diaphragm early during tumor development and restored to normal by 4 weeks but is unaffected in quadriceps.

**Figure 9:**
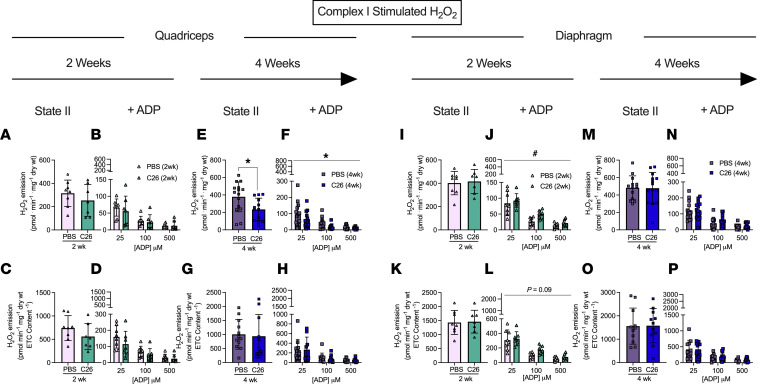
Complex I–stimulated mH_2_O_2_ emission in quadriceps and diaphragm muscle of C26 tumor–bearing mice. At 2 and 4 weeks, quadriceps mH_2_O_2_ emission supported by pyruvate (10 mM) and malate (2 mM) (NADH) was assessed under maximal state II (no ADP) conditions in the presence of 20 mM creatine (**A** and **E**) and under a range of [ADP] to model metabolic demand (**B** and **F**). These measures were also normalized to total ETC subunit content (**C**, **D**, **G**, and **H**) to permit comparisons of intrinsic mitochondrial respiratory responses in each group. These measures were repeated in the diaphragm (**I**–**P**). Results represent mean ± SD; *n* = 8–16; 2-tailed *t* tests were used to determine the difference between PBS(2wk) versus C26(2wk) and PBS (4wk) versus C26(4wk) for state II H_2_O_2_ emission. A 2-way ANOVA was used to determine the difference between PBS(2wk) versus C26(2wk) and PBS (4wk) versus C26(4wk). ^#^*P* < 0.05, PBS(2wk) versus C26(2wk); *P < 0.05, PBS(4wk) versus C26(4wk).

We stimulated complex I with pyruvate (10 mM) and malate (2 mM) to generate NADH in the absence of ADP to elicit mH_2_O_2_ emission and determined ADP’s ability to attenuate this emission as occurs naturally during oxidative phosphorylation (see schematic representation, Figure 6). At 2 weeks following C26 implantations, quadriceps mH_2_O_2_ emission was similar to PBS controls under maximal emission conditions (no suppression by ADP, state II; Figure 9, A and C) and during suppression by ADP (Figure 9, B and D). By 4 weeks of C26 growth, quadriceps mH_2_O_2_ was lower than PBS in both maximal and ADP-suppressive states (Figure 9, E and F). However, when mH_2_O_2_ was normalized to total ETC subunit content, no differences were observed between C26 and PBS (Figure 9, G and H). This finding suggests eventual decreases in quadriceps mH_2_O_2_ by 4 weeks were related to decreased ETC subunit content as shown in Figure 4. Due to limited tissue availability, pyruvate-supported mH_2_O_2_ was assessed only in the +creatine condition.

In contrast to the lower mH_2_O_2_ in quadriceps, diaphragm mH_2_O_2_ was greater in C26 mice at 2 weeks relative to PBS in the presence of ADP despite no change in maximal mH_2_O_2_ (Figure 9, I and J). This finding reveals C26 induces early elevations in diaphragm mH_2_O_2_ that are likely due to a specific impairment in the ability of ADP to attenuate H_2_O_2_ emission. Moreover, when mH_2_O_2_ emission was normalized to total ETC subunit content at 2 weeks, maximal mH_2_O_2_ emission remained unchanged (Figure 9K), while the higher emissions in the presence of ADP did not reach significance (Figure 9L) but mirrored patterns observed when normalized to wet mass of tissue as noted above. At 4 weeks, there were no differences in diaphragm mH_2_O_2_ under maximal or submaximal (presence of ADP) conditions using either normalization approach (Figure 9, M–P), suggesting diaphragm mitochondria are plastic and can eventually restore mH_2_O_2_ to normal levels.

Succinate-supported mH_2_O_2_ emission generally did not change in either muscle in C26 versus PBS at either time point (Supplemental Figure 4). This finding suggests reverse electron flux to complex I from complex II (23) was not altered by C26 cancer, and the responses mentioned above using pyruvate/malate reveal a specific alteration in mH_2_O_2_ emission supported by forward electron flux through complex I.

### Inhibition of mitochondrial ATP synthase directly impairs force production in diaphragm.

**Figure 10:**
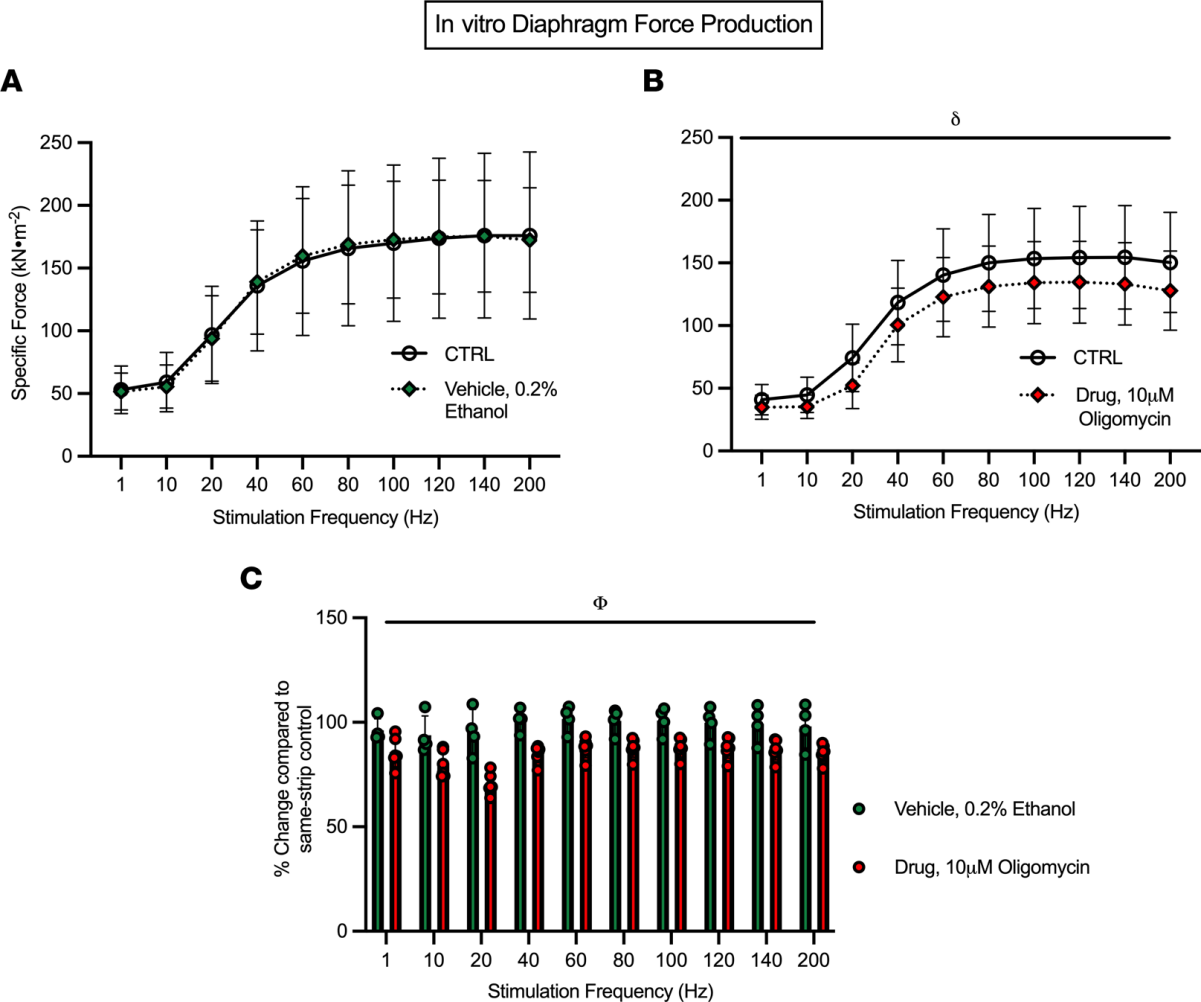
Inhibition of mitochondrial oxidative phosphorylation acutely lowers in vitro diaphragm force production. Diaphragm strips underwent an in vitro force-frequency protocol (CTRL) and were then incubated with 0.2% ethanol (vehicle) while relaxing for 40 minutes followed by a second assessment (**A**, *n* = 4). A similar approach was used with 10 μM oligomycin in separate strips (**B**, *n* = 6). The relative change in force production in both vehicle and oligomycin conditions compared with their respective CTRL was also analyzed (**C**, *n* = 4–6). Results represent mean ± SD; a 2-way ANOVA was used to determine the difference between CTRL versus vehicle, CTRL versus drug, and vehicle versus drug. *n* = 4–6; ^δ^*P* < 0.05, CTRL versus drug; ^Φ^*P* < 0.05, vehicle versus drug.

These observations suggest that mitochondrial stress contributes to early weakness at 2 weeks in both muscles and that restoration of force in quadriceps by 4 weeks is related to a natural compensatory increase in mitochondria respiratory control. In order to establish a direct link between mitochondria and force production, we designed an in vitro experiment whereby we used oligomycin to inhibit ATP synthase in diaphragm strips from healthy CD2F1 mice. Within each strip, force-frequency relationships were assessed in standard assay media (CTRL) (Figure 10A). Strips were then incubated with 0.2% ethanol (vehicle) while relaxing in the apparatus for 40 minutes. A second force-frequency assessment was then performed, which demonstrated no effect of the vehicle (Figure 10A). We then repeated this experimental design on separate muscle strips but in the presence of 10 μM oligomycin during the 40-minute incubation after a control stimulation. There was a main effect whereby oligomycin lowered force production (Figure 10B). Likewise, a main effect of lower force was exhibited within the oligomycin condition when the change in force production relative to control strips was analyzed (Figure 10C).

### Reduced, oxidized, and total glutathione are decreased in quadriceps after 4 weeks of tumor bearing despite an increase in the reduced/oxidized glutathione ratio.

**Figure 11:**
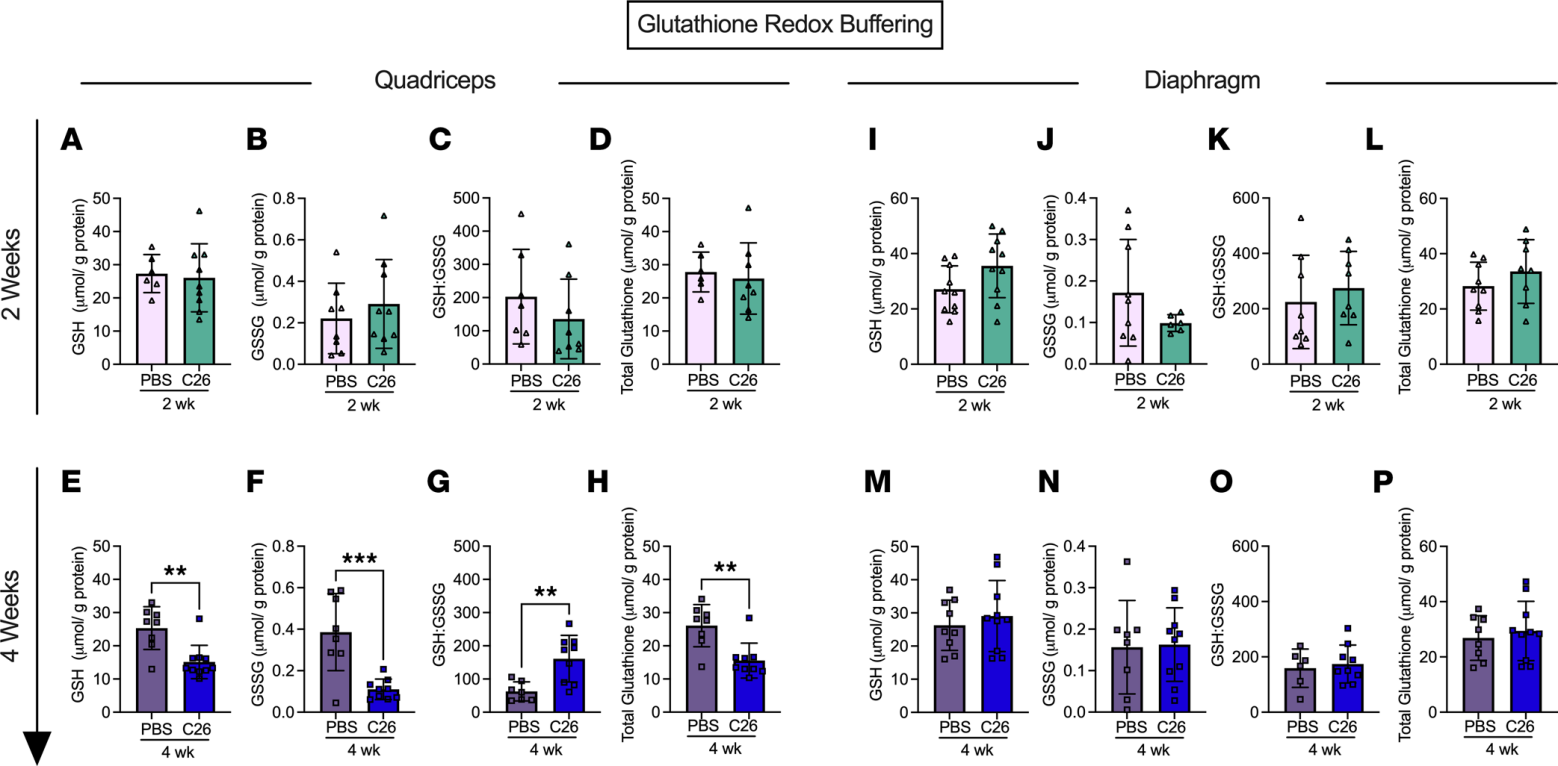
Glutathione redox buffering in quadriceps and diaphragm muscle of C26 tumor–bearing mice. Reduced glutathione was measured in the quadriceps of the 2- and 4-week cohort (**A** and **E**) along with oxidized glutathione (**B** and **F**). The ratio of reduced to oxidized glutathione was also analyzed (**C** and **G**) along with the total glutathione (**D** and **H**). This was repeated in the diaphragm (**I**–**P**). Results represent mean ± SD; *n* = 8–10; 2-tailed *t* tests were used to determine the difference between PBS(2wk) versus C26(2wk) and PBS (4wk) versus C26(4wk). **P < 0.01 PBS(4wk) versus C26(4wk); ***P < 0.001 PBS(4wk) versus C26(4wk).

**Figure 12:**
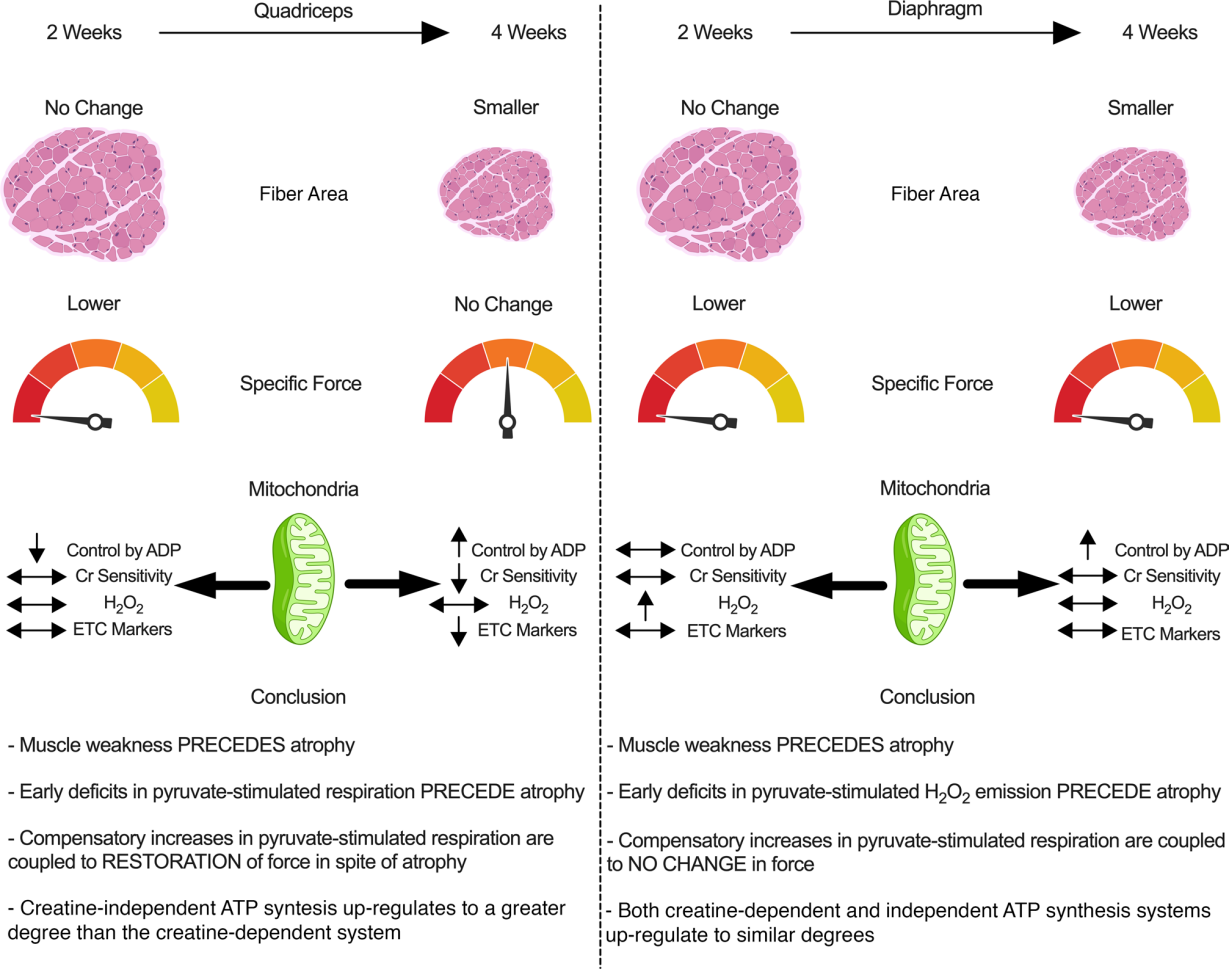
Summary of the time-dependent and muscle-specific adaptations to C26 xenografts in CD2F1 mice. At 2 weeks, early impairments in force-generating capacity are associated with reductions in mitochondrial pyruvate/malate-supported, ADP-stimulated respiration in quadriceps and elevated mH_2_O_2_ emission in diaphragm. These distinct mitochondrial responses precede atrophy in both muscles by 4 weeks. At this time, quadriceps and diaphragm responses to C26 become heterogeneous. The restoration of force-generating capacity in quadriceps in spite of atrophy is not observed in the diaphragm even though both muscles demonstrate apparent compensatory increases in mitochondrial ADP-stimulated respiration. The mitochondrial responses to cancer are more diverse in quadriceps than diaphragm, with increases in respiration by 4 weeks occurring as a potential compensation for reductions in mitochondrial ETC markers. Mitochondrial creatine metabolism is impaired in quadriceps by 4 weeks. Downward arrow represents decrease; upward arrow represents increase; double-headed arrow represents no change. Created with BioRender.com.

Noting the mH_2_O_2_ patterns were also time- and muscle-dependent, we then determined whether there was a corresponding impact on cellular redox state by assessing the equilibrium of the glutathione redox buffer. At 2 weeks, there were no differences in reduced glutathione (GSH), oxidized glutathione (GSSG), GSH/GSSG ratio, and total glutathione in the either muscle (Figure 11, A–D and I–L). At 4 weeks, quadriceps demonstrated a lower content of reduced, oxidized, and total glutathione, but interestingly there was an increase in the GSH/GSSG ratio (Figure 11, E–H). No changes in any measure were observed in diaphragm at 4 weeks. (Figure 11, M–P). A summary of the time-dependent and muscle-specific adaptations to C26 cancer is provided in Figure 12.

## Discussion

Certain indices of skeletal muscle mitochondrial stress have been associated with cancer cachexia in various mouse models (11–13, 24, 25), but the time- and muscle-dependent relationship remains unclear. Moreover, the early relationships between force production and atrophy have not been understood. Here, we demonstrate how quadriceps and diaphragm have both common and distinct time-dependent responses to cancer in the C26 colon carcinoma mouse model of cancer cachexia. First, a reduction in specific muscle force was observed prior to atrophy in both muscles, yet an eventual increase in specific force production to control levels by 4 weeks occurred only in quadriceps. This restoration of specific force indicates an apparent intrinsic compensation given absolute force was lower at this later time point due to atrophy. Second, atrophy in most fiber types was preceded by altered mitochondrial bioenergetics, but the individual relationship differed between muscles with decreases in respiration occurring in quadriceps in contrast to elevated mH_2_O_2_ emission in diaphragm. Third, both muscles upregulated mitochondrial respiration supported specifically by pyruvate and malate substrates at 4 weeks, which may reflect a hormetic adaptation to maintain energy homeostasis during cachexia. Likewise, the diaphragm restored complex I–supported mitochondrial H_2_O_2_ emission to normal lower levels by 4 weeks, which demonstrates the transient nature of this potential redox pressure.

Collectively, these findings suggest muscle weakness can occur before atrophy during C26 cancer, and this progression is related to dynamic time-dependent changes in mitochondrial bioenergetics that are unique to each muscle.

### Mitochondrial bioenergetic alterations and skeletal muscle force reductions precede skeletal muscle atrophy.

Work from Brown et al. suggested mitochondrial degradation and dysfunction precedes muscle atrophy in the LLC xenograft mouse model of cancer cachexia (12). In this study, atrophy markers occurred after earlier indices of mitochondrial degeneration in comparator muscles (flexor digitorum brevis and plantaris), including mitochondrial degradation, respiratory control ratios, and H_2_O_2_ emission. The findings of the present study support this proposal with a comparison of atrophy, mitochondrial respiration, and mH_2_O_2_ emission within the same muscle types, namely quadriceps and diaphragm. These findings also extend the proposal by showing muscle-specific mitochondrial alterations occur concurrent to muscle weakness and before atrophy. Specifically, early decreases in respiratory kinetics in quadriceps were not seen in diaphragm, suggesting more oxidative muscle might avoid such respiratory decrements. Conversely, early increases in mH_2_O_2_ emission seen in diaphragm did not occur in quadriceps. These relationships suggest targeted therapies to counter mitochondrial alterations during cancer cachexia could consider the specific bioenergetic function that is altered at precise time points in each muscle type.

This relationship between early mitochondrial stress prior to atrophy in both muscles becomes further complicated when considering force production. Muscle weakness occurred at 2 weeks in both muscles before atrophy, which highlights a shared pattern in the progression of muscle dysfunction during cancer. While the purpose of this investigation was not to address other mechanisms regulating force production, reduced fiber sizes cannot be an explanation given atrophy did not occur until after weakness was first observed. In this regard, the reduction in absolute force in quadriceps at 2 weeks was therefore due to the lower specific force. However, the distinct mitochondrial signatures in both muscles at 2 weeks could guide additional questions. For example, in the quadriceps, the early reductions in specific force were associated with early decreases in mitochondrial respiratory control by ADP. When specific force production was restored to control levels by 4 weeks, respiration actually increased above control levels when normalized to ETC subunit content. This dynamic relationship is intriguing and suggests early quadriceps weakness might be due to impairments in mitochondrial energy provision that is nonetheless plastic and capable of adapting — possibly as a hormetic response to the earlier respiratory deficiency — to correct this weakness through super-compensations in energy supply.

In contrast, the diaphragm weakness seen at 2 weeks might be linked to an early redox pressure given elevated mH_2_O_2_ was observed. This observation is consistent with prior observations of early and transient increases in H_2_O_2_ emission in diaphragm in the LLC mouse model of cancer cachexia (24). However, these changes in mH_2_O_2_ did not alter glutathione in reduced or oxidized forms or in the static measure of GSH/GSSG equilibrium. While these common measures of glutathione redox buffering are often used to interpret changes in the redox proteome (26), the measures do not necessarily capture the kinetic aspect by which GSH is recycled through glutathione reductases and glutathione peroxidases. As such, a lack of change in these variables does not rule out a signaling role for H_2_O_2_ in mediating the diaphragm’s adaptive response as one possibility may be that the diaphragm was capable of sustaining greater glutathione recycling rates. These lysate-based measures also do not rule out changes in glutathione-mediated thiol oxidation in specific subcompartments of the cell, including mitochondrial or nuclear fractions (26–28). Likewise, mitochondrial H_2_O_2_ can also oxidize peroxiredoxins through a thioredoxin-mediated recycling mechanism, the relevance of which to muscle adaptation during diseases is only now being unravelled in more detail (29). Consideration of such complexities in redox signaling, much of which is H_2_O_2_ dependent, demonstrates how no single assay can capture the impact of mH_2_O_2_ on redox signaling–mediated adaptive responses yet exemplifies the considerable research required to further explore the impact of the mH_2_O_2_ relationships to cancer cachexia reported in this and other studies (12). Last, the degree to which mH_2_O_2_ influences mitochondrial respiratory control must also be considered in greater depth. For example, the diaphragm did not show reductions in respiration at 2 or 4 weeks yet demonstrated an unexpected increase in respiration at 4 weeks. The explanation for this increase is not apparent but might suggest an earlier energetic deficiency occurred outside of our selected time points. Alternatively, the use of pyruvate as a glucose-derived substrate to stimulate respiration does not rule out a possible change in substrate selection that may be occurring as part of grander adaptive processes. Future studies could compare both pyruvate and fatty acid substrates to determine if such responses are ubiquitous across all substrate oxidation pathways.

The mechanisms by which mitochondria contribute to weakness and atrophy continue to be an area of considerable research and cannot be resolved by a single investigation. However, we attempted to resolve whether mitochondrial stress can acutely suppress force production using an in vitro approach that is removed from potential systemic responses to cancer that might also contribute to weakness. As demonstrated in Figure 10, acute treatment of diaphragm muscle with the ATP synthase inhibitor oligomycin decreased specific force production. Oligomycin inhibits ATP synthesis but theoretically may also increase mitochondrial ROS through increased membrane potential–related electron slip (23). As this cannot be verified during contraction in vitro using our system, the findings nonetheless demonstrate that mitochondrial stress can acutely depress specific force production. This observation matches previous findings of various mitochondrial inhibitors on single-fiber contraction performed in vitro (30). Likewise, in cultured myotubes, a mitochondria-targeted antioxidant attenuated cisplatin-induced (chemotherapy) atrophy by preventing increases in mitochondrial ROS (31). Similarly, a mitochondria-targeted peptide administered to the same C26 cancer cachexia model used in the present study preserved diaphragm force production and fiber cross-sectional area by preventing changes in indices of mitochondrial ATP synthesis and ROS (13). By comparing this prior literature to the time-dependent and muscle-specific responses to C26 cancer in the present study, the dynamic relationship between weakness, atrophy, and mitochondrial stress–specific responses could now be explored with such mitochondrial enhancing compounds using a design that captures time-dependent responses across muscle types.

### Perspectives on the potential for mitochondrial hormesis in quadriceps and diaphragm.

The findings of lower respiration and increased mH_2_O_2_ emission at 2 weeks are consistent with prior reports at various time points and muscle in the LLC, C26, and peritoneal carcinosis mouse models (11–13, 24, 25). To our knowledge, the eventual increase in pyruvate-supported respiration seen in both quadriceps and diaphragm in the present study is novel, whereas the attenuation of mH_2_O_2_ emission seen in the diaphragm is consistent with past reports in the plantaris (12) and diaphragm (24) in the LLC mouse model of cachexia. As noted above, mitochondrial respiratory control by ADP increased above control in both muscles despite stress being observed earlier only in quadriceps. We questioned whether this early reduction in respiration represented an energy crisis triggering compensatory signaling through the energy sensor AMPK — a pathway that triggers compensatory mitochondrial biogenesis or upregulation of substrate-specific oxidation (32). We did not observe an effect of cancer on AMPK phosphorylation at either time point (Supplemental Figure 3), though there was a trend in the quadriceps at 4 weeks of tumor bearing whereby AMPK content was increased. Nonetheless, these results do not rule out the potential for AMPK activation at other time points or a more sustained phosphorylation of downstream AMPK targets. There are also multiple feedback control systems linking metabolic stress to respiratory control that are independent of AMPK, which might be considered in future investigations (33).

The design of substrate titration protocols lends insight into the specific mechanisms by which respiration and mH_2_O_2_ become altered during cancer. For example, as pyruvate/malate were used as the substrates to generate NADH to stimulate complex I–supported respiration, future investigations might consider the potential for cancer to upregulate complex I or pyruvate dehydrogenase activity, though maximal activity given saturating pyruvate concentrations was used. Also, the consistent increase in respiration across a wide spectrum of ADP concentrations by 4 weeks in both muscles suggests mitochondrial responsiveness to a wide range of metabolic demands may have been enhanced such that key regulators of matrix ADP/ATP cycling could be considered for future directions (Figure 6). ADP was also more effective at attenuating mH_2_O_2_ (23) in quadriceps by 4 weeks (Figure 9), which supports this possibility. Collectively, these findings suggest cancer disrupts mitochondrial bioenergetics by specifically desensitizing mitochondria to ADP in both muscles.

Mitochondrial creatine metabolism appeared to be less capable of adapting in quadriceps by 4 weeks (Figure 7L), suggesting mtCK-dependent phosphate shuttling was more affected in this muscle than diaphragm, which showed no such deficiency. In fact, the creatine-independent (-creatine) system showed homogeneous plasticity by upregulating in both muscles by 4 weeks while the creatine-dependent system upregulated only in the diaphragm. These findings suggest mitochondrial creatine metabolism may be disrupted in quadriceps muscle during cancer, which may impact energy homeostasis given the importance of this system in certain muscles (21).

In general, the diaphragm appeared to be superior to quadriceps with respect to maintaining mitochondrial ETC content markers and respiratory control by ADP at 2 weeks, with evidence of super-compensation in respiratory function at 4 weeks. Furthermore, reductions in ETC protein contents were observed in quadriceps at 4 weeks after C26 implantation whereas no changes were observed in diaphragm. This resilience of diaphragm appears to be a unique finding given prior reports have also shown lower mitochondrial protein markers from various pathways and muscle types in LLC and *APC*^(Min/+)^ mouse models of cancer cachexia (10, 12). While the mechanisms for this muscle heterogeneity remain unclear, one possibility relates to muscle contractile activity. The diaphragm constantly contracts in vivo whereas quadriceps is used during locomotion. As mitochondrial content and substrate oxidation are regulated by contractile activity (33), future directions might consider whether the diaphragm holds a special mitochondrial “resistance” to cancer with respect to energy homeostasis, which might support the growing notion of chronic contractile activity in improving muscle health during cancer (34).

In conclusion, this investigation reports muscle weakness precedes atrophy in quadriceps and diaphragm in the C26 colon carcinoma mouse model of cancer cachexia. This progression was associated with heterogenous muscle-specific and time-dependent mitochondrial responses in both muscles. Specifically, an early energetic stress (reduced respiratory control by ADP) was more apparent in quadriceps in contrast to a mitochondrial redox pressure in diaphragm. These early mitochondrial stressors were seemingly corrected as cancer progressed despite the development of atrophy in both muscles and a unique increase in specific force production in quadriceps. This apparent intrinsic compensation in quadriceps may have partially mitigated the contribution of atrophy to the reduced absolute force seen at 4 weeks. Moreover, C26 cancer caused a unique impairment in the coupling of the mtCK system to ATP generation in quadriceps whereas this system was not affected in diaphragm. This dynamic plasticity across time demonstrates how the effects of cancer on one muscle may not predict the response in another muscle type. The findings also highlight how understanding heterogeneity may identify mechanisms that determine whether a given muscle might be sensitive, or resistant, to cancer cachexia.

## Methods

### Animal care

We purchased 48 eight-week-old male CD2F1 mice from Envigo. Upon arrival, mice were housed and given a minimum of 72 hours to acclimatize before cancer implantations. All mice were provided access to standard chow and water ad libitum. While food intake was not measured, reductions in food intake reported in this model of cachexia did not contribute to reduced muscle weights, fiber CSA, or muscle force given pair-fed mice retained normal muscle parameters compared to C26 mice (35, 36). Mice were monitored daily for general well-being, tumor ulcerations, and tumor size. If mice had demonstrated signs of extreme distress, mice would have been sacrificed as soon as possible; however, this was never required.

### C26 cell culture and tumor implantation

C26 cancer cells (purchased from NIH National Cancer Institute) were plated at passage 2–3 in T-75 flasks in DMEM supplemented with 10% FBS plus 1% penicillin and streptomycin. Once confluent, cells were trypsinized, counted, and diluted in PBS. C26 cells (5 × 10^5^) suspended in 100 μL sterile PBS were implanted subcutaneously to both flanks of mice at 8 weeks of age (11). For control, mice received identical subcutaneous injections of 100 μL sterile PBS and aged for 2 weeks [PBS (2wk); *n* = 8] and 4 weeks [PBS (4wk); *n* = 16]. Tumors developed for 14–17 days [C26 (2wk); *n* = 8] and 26–29 days [C26 (4wk); *n* = 16]. Tumors were measured daily, recording the length and width of tumors with digital calipers using the following formula to obtain tumor volume (volume of a sphere): 4/3 × π × (length/2) × (width/2)^2^, in accordance with York University Animal Care Committee guidelines. The same investigator was responsible for measuring tumor sizes throughout the study as preliminary work demonstrated that tumor size measurements can vary between individuals (data not shown; coefficient of variation 7.2% between 3 individuals, coefficient of variation 1.3% within designated individual).

### Surgery procedure

Quadriceps, soleus, plantaris, gastrocnemius, tibialis anterior, extensor digitorum longus, and spleen were quickly collected under isoflurane anesthesia prior to euthanasia. Tissues were weighed and snap-frozen in liquid nitrogen and stored at –80°C. Quadriceps and diaphragm muscles were placed in BIOPS containing (in mM) 50 MES Hydrate, 7.23 K_2_EGTA, 2.77 CaK_2_EGTA, 20 imidazole, 0.5 dithiothreitol, 20 taurine, 5.77 ATP, 15 PCr, and 6.56 MgCl_2_·6 H_2_O (pH 7.1) to be prepared for mitochondrial bioenergetic assays. Quadriceps from 1 leg and diaphragm strips were harvested for mitochondrial bioenergetic assays while the quadriceps from the contracted leg and a separate diaphragm strip were used for force measurements. The diaphragm strip used for force measurements was cut within 30 seconds of the entire diaphragm being placed in BIOPS prior to transferring the strip to Ringer’s solution as noted below.

### In situ quadriceps force and in vitro diaphragm force

In situ force production for quadriceps muscle was partially adapted from previous literature (37). Mice were anesthetized with isoflurane and shaved of all hair on their hind limb. An incision was made above the patella to expose the femoral tendon, which was then tightly secured with suture. Once the knot was in place, the tendon was carefully severed, and the suture was attached to an Aurora Scientific 305C muscle lever arm with a hook. The knee was secured with a vertical knee clamp, immobilizing the knee joint with a 27G needle. Contraction of the quadriceps was controlled through percutaneous stimulation of the femoral nerve anterior to the hip joint. Optimal resting length (L_o_) was determined using single twitches (pulse width = 0.2 ms) at varying muscle lengths. Once L_o_ was established, force as a function of stimulation frequency was measured during 8 isometric contractions at varying stimulation frequencies (1, 20, 40, 60, 80, 100, 120, 140 Hz). The quadriceps muscle was then weighed and used for normalization of force production (“specific force”).

In vitro force production for diaphragm muscle was partially adapted from previous literature (38). Briefly, the diaphragm strip used for force production was placed in a petri dish of approximately 25°C Ringer’s solution containing (in mM): 121 NaCl, 5 KCl, 1.8 CaCl_2_, 0.5 MgCl_2_ 0.4 NaHPO_4_, 24 NaHCO_3_, 5.5 glucose, and 0.1 EDTA; pH 7.3; oxygenated with 95% O_2_ and 5% CO_2_. Diaphragm strips were cut from the central region of the lateral costal hemidiaphragm. Silk suture was tied to the central tendon as well the ribs, and the preparation was transferred to an oxygenated bath filled with Ringer’s solution, maintained at 25°C. The suture secured to the central tendon was then attached to a lever arm while the suture loop secured to the ribs was attached to a force transducer. The diaphragm strip was situated between flanking platinum electrodes driven by a biphasic stimulator (model 305C; Aurora Scientific, Inc.). Optimal L_o_ was determined using twitches (pulse width = 0.2 ms) at varying muscle lengths. Once L_o_ was established, force as a function of stimulation frequency was measured during 10 isometric contractions at varying stimulation frequencies (1, 10, 20, 40, 60, 80, 100, 120, 140, 200 Hz). Force production was normalized (“specific force”) to the calculated CSA of the muscle strip (m/l×d, where m is the muscle mass, l is the length, and d is mammalian skeletal muscle density: 1.06 mg/mm^3^). Absolute force for whole muscle was reported for quadriceps but not diaphragm given the latter was assessed with muscle strips.

### Mitochondrial bioenergetic assessments

#### Preparation of permeabilized muscle fibers.

The assessment of mitochondrial bioenergetics was performed as described previously in our publications (17–19). Briefly, the quadriceps and diaphragm from the mouse were removed and placed in BIOPS. Muscle was trimmed of connective tissue and fat and divided into small muscle bundles (~1.2–3.7 mg wet weight for quadriceps and 0.6–2.1 mg for diaphragm). Each bundle was gently separated along the longitudinal axis to form bundles that were treated with 40 μg/mL saponin in BIOPS on a rotor for 30 minutes at 4°C. Following permeabilization, the permeabilized muscle fiber bundles (PmFBs) for respiration assessments were blotted and weighed in about 1.5 mL of tared prechilled BIOPS (muscle-relaxing media) to ensure PmFBs remained relaxed and hydrated rather than exposed to open air. Wet weights were used given small pieces of muscle can detach during respirometry assessments, albeit greatly reduced by blebbistatin (described below). Mean ± SEM wet weights (mg) were 2.4 ± 0.07 for quadriceps and 1.3 ± 0.04 for diaphragm. The remaining PmFBs for mH_2_O_2_ were not weighed at this step as these data were normalized to fully recovered dry weights taken after the experiments. All PmFBs were then washed in MiRO5 on a rotator for 15 minutes at 4°C to remove the cytoplasm. MiRO5 contained (in mM) 0.5 EGTA, 10 KH_2_PO_4_, 3 MgCl_2_·6 H_2_O, 60 K-lactobionate, 20 HEPES, 20 taurine, and 110 sucrose and 1 mg/mL fatty acid–free BSA (pH 7.1).

#### Mitochondrial respiration.

High-resolution O_2_ consumption measurements were conducted in 2 mL of respiration medium (MiRO5) using the Oxygraph-2k (Oroboros Instruments, Corp.) with stirring at 750 rpm at 37°C. MiRO5 contained 20 mM creatine to saturate mtCK and promote phosphate shuttling through mtCK or was kept void of creatine to prevent the activation of mtCK (39) as described in Figure 6. For ADP-stimulated respiratory kinetics, our previously published procedures to stimulate complex I– and II–supported respiration were employed (17–19). We added 5 mM pyruvate and 2 mM malate as complex I–specific substrates (via generation of NADH to saturate electron entry into complex I) followed by a titration of submaximal ADP (25, 100, and 500 μM) and maximal ADP (up to 5,000 μM in the presence of creatine or 30,000 μM in the absence of creatine). 25 μM and 100 μM are close to low and high points of previous estimates of free ADP concentrations in human skeletal muscle in resting and high-intensity exercise states and therefore allow the determination of mitochondrial responsiveness to a physiological spectrum of low to high energy demands (40–44). Saturating [ADP] were different depending on the muscle and presence or absence of creatine in the experimental media. Mitochondrial respiration was normalized to mass of fiber bundles as well as total content of ETC subunits detected with the antibody cocktail listed below. This normalization was performed on a separate piece of the same muscle from the same mouse to evaluate whether changes in respiration per mass were due to alterations in mitochondrial content or intrinsic mitochondrial respiratory responses. While normalizing bioenergetic fluxes to ETC content markers within the same bundle would theoretically reduce variability, the occasional separation of 1–2 fibers in chambers after an experiment may cause underestimations of total bundle ETC marker contents.

Kmapp for creatine to ADP was not established as we have observed that many permeabilized fibers from past studies do not fit Michaelis-Menten kinetics with these assay conditions (low to modest *R*^2^). Creatine accelerates matrix ADP/ATP cycling at submaximal [ADP] and lowers the Kmapp for ADP in some muscles (21, 33). Therefore, in order to evaluate mitochondrial creatine sensitivity, 100 and 500 μM ADP were used to calculate a creatine sensitivity index. Following the ADP titration, cytochrome *c* was added to test for mitochondrial membrane integrity. Finally, succinate (20 mM) was then added to saturate electron entry into complex II.

All experiments were conducted in the presence of 5 μM blebbistatin (BLEB) in the assay media to prevent spontaneous contraction of PmFBs, which has been shown to occur in response to ADP at 37°C that alters respiration rates (39, 45). Polarographic oxygen measurements were acquired in 2-second intervals with the rate of respiration derived from 40 data points and expressed as pmol/s/mg wet weight. PmFBs were weighed in about 1.5 mL of tared BIOPS to relax muscle as noted above.

#### mH_2_O_2_.

mH_2_O_2_ was determined spectrofluorometrically (QuantaMaster 40, HORIBA Scientific) in a quartz cuvette with continuous stirring at 37°C, in 1 mL of Buffer Z supplemented with 10 μM Amplex Ultra Red, 0.5 U/mL horseradish peroxidase, 1 mM EGTA, 40 U/mL Cu/Zn-SOD1, 5 μM BLEB, and 20 mM Cr. Buffer Z contained (in mM) 105 K-MES, 30 KCl, 10 KH_2_PO_4_, 5 MgCl_2_ · 6H_2_O, and 1 EGTA and 5 mg/mL BSA (pH 7.4). State II mH_2_O_2_ (maximal emission in the absence of ADP) was induced using the complex I–supporting substrates (NADH) pyruvate (10 mM) and malate (2 mM) to assess maximal (state II, no ADP) mH_2_O_2_ as described previously (18). Following the induction of state II mH_2_O_2_, a titration of ADP was employed to progressively attenuate mH_2_O_2_ as occurs when membrane potential declines during oxidative phosphorylation (Figure 6). After the experiments, the fibers were rinsed in double-deionized H_2_O, lyophilized in a freeze dryer (Labconco) for more than 4 hours, and weighed on a microbalance (Sartorius Cubis Microbalance). The rate of mH_2_O_2_ emission was calculated from the slope (F/min) using a standard curve established with the same reaction conditions and normalized to fiber bundle dry weight.

### Western blotting

A frozen piece of quadriceps and diaphragm from each animal was homogenized in a plastic microcentrifuge tube with a tapered Teflon pestle in ice-cold buffer containing (mm) 10 Tris/HCl, 150 NaCl, 1 EDTA, 1 EGTA, 2.5 Na_4_O_7_P_2_, and 1 Na_3_VO_4_ and 1% Triton X-100 and PhosSTOP inhibitor tablet (MilliporeSigma) (pH 7.0) as published previously (46). Protein concentrations were determined using a bicinchoninic acid assay (Life Technologies, Thermo Fisher Scientific). A total of 15–30 μg of denatured and reduced protein was subjected to 10%–12% gradient SDS-PAGE followed by transfer to low-fluorescence polyvinylidene difluoride membrane. Membranes were blocked with Odyssey Blocking Buffer (LI-COR) and immunoblotted overnight (4°C) with antibodies specific to each protein. A commercially available monoclonal antibody was used to detect ETC proteins (rodent OXPHOS Cocktail, ab110413; Abcam, 1:250 dilution), including V-ATP5A (55 kDa), III-UQCRC2 (48 kDa), IV-MTCO1 (40 kDa), II-SDHB (30 kDa), and I-NDUFB8 (20 kDa). Commercially available polyclonal antibodies were used to detect AMPKα (rabbit, Cell Signaling Technology, 2532; 62 kDa; 1:1,000) and p-AMPKα Thr172 (rabbit, Cell Signaling Technology, 2535, 62 kDa; 1:500) as used previously (46).

After overnight incubation in primary antibodies, membranes were washed 3 times for 5 minutes in TBS-Tween and incubated for 1 hour at room temperature with the corresponding infrared fluorescent secondary antibody (LI-COR IRDye 680RD 925-68020 or 800CW 925-32214) at a dilution previously optimized (1:20,000). Immunoreactive proteins were detected by infrared imaging (LI-COR CLx; LI-COR) and quantified by densitometry using ImageJ (NIH). All images were normalized to Amido Black total protein stain (A8181, MilliporeSigma) using the entire lane corresponding to each sample.

### Immunofluorescence analysis

Quadriceps (cut longitudinally to include half the vastus intermedius and full vastus lateralis) and diaphragm muscle samples embedded in O.C.T medium (Thermo Fisher Scientific) were cut into 10 μm sections with a cryostat (HM525 NX, Thermo Fisher Scientific) maintained at –20°C. Muscle fiber type was determined as previously described (47), with minor modifications. All primary antibodies were purchased from the Developmental Studies Hybridoma Bank (University of Iowa), and secondary antibodies were purchased from Invitrogen, Thermo Fisher Scientific. Briefly, slides were blocked with 5% goat serum (MilliporeSigma) in PBS for 1 hour at room temperature. Next, slides were incubated with primary antibodies against MHC I (BA-F8; 1:25), MHC IIA (SC-71; 1:1,000), and MHC IIB (BF-F3; 1:50) for 2 hours at room temperature. Afterward, slides were washed 3 times in PBS for 5 minutes and then incubated with secondary antibodies (MHC I; Alexa Fluor 350 IgG2b; 1:1,000) (MHC IIa; Alexa Fluor 488 IgG1; 1:1,000) (MHC IIb; Alexa Fluor 568 IgM; 1:1,000) for 1 hour at room temperature. Slides were then washed 3 times in PBS for 5 minutes and mounted with ProLong antifade reagent (Life Technologies, Thermo Fisher Scientific). Images were acquired the next day using EVOS FL Auto 2 Imaging System (Invitrogen, Thermo Fisher Scientific). Individual images were taken across the entire cross section and then assembled into a composite image. A total of 20–30 muscle fibers per fiber type were selected randomly throughout the cross section and traced with ImageJ software to assess CSA after calibrations with a corresponding scale bar. Muscle fibers that appeared black were recorded as MHC IIX (47).

### Real-time reverse transcription PCR analyses of mRNA

Measurement of mRNA was completed as previously described (48). Briefly, frozen tissues were homogenized with TRIzol (Life Technologies, Thermo Fisher Scientific, catalog 15596026) and RNA was isolated with a commercial kit. RNA concentrations were quantified with a Take3 microvolume microplate with a PowerWave XS microplate reader (BioTek Instruments Inc.). RNA was only used if the sample was of sufficient quality (260/280 ratio > 2). Isolated RNA was then reverse-transcribed into cDNA using commercial reagents (Superscript Vilo, catalog 11755500; Life Technologies, Thermo Fisher Scientific). cDNA was then serially diluted to 1:100 and used for quantitative real-time PCR analysis. cDNA was combined with appropriate probes and master mix and amplified in a reaction of 40 cycles of denaturation, annealing, and extension at 95, 60, and 72°C. Data were quantified with the ΔΔCt method. Data were normalized to control groups. 18s was used as an internal housekeeping gene (clone Mm03928990_g1) and did not differ between groups. Probes of interest included Atrogin/FBXO32 (clone Mm00499523_m1), Murf1/Trim63 (clone Mm01185221_m1), Gadd45a (clone Mm00432802_m1), and Ubc (clone Mm02525934_g1).

### Glutathione analysis

Glutathione was measured as previously published (18, 20). Briefly, GSH and GSSG were measured using the Shimadzu Nexera X2 UHPLC system (Mandel Scientific). Quadriceps and diaphragm were homogenized in 50 mM Tris buffer with 10 mM boric acid, 2 mM l-serine, 20 μM acivicin, and 5 mM *N*-ethylamide and acidified using TCA (for GSH) and PCA (for GSSG). Samples were separated with a Zorbax C18 column (Agilent Technologies). GSH was assessed by UV-HPLC (265 nm wavelength) monitoring of NEM-GSH using a 0.25% glacial acetic acid mobile phase with 6% acetonitrile at 1.05 mL/min flow rate detected at 265 nm. GSSG was assessed by fluorescent HPLC (excited at 350 nm and detected at 420 nm emission) using HPLC/UHPLC fluorescence detector (Mandel Scientific). GSSG samples were diluted in 0.5 M NaOH and run using 25 mM Na_2_HPO_4_ in HPLC-grade water with 15% methanol mobile phase at a 0.5 mL/min flow rate by tracking *o*-pthalymide–tagged GSH.

### Statistics

Results are expressed as mean ± SD. The level of significance was established at *P* < 0.05 for all statistics. The D’Agostino-Pearson omnibus normality test was first performed to determine whether data resembled a Gaussian distribution, and all data were subject to the ROUT test to identify and exclude outliers. Western blot results for proteins in the ETC subunit complexes I, IV, and V in quadriceps failed normality as did proteins in complexes I, II, IV, and V for diaphragm. In addition, quadriceps and diaphragm Δ-glutamate respiration failed normality and were analyzed using a nonparametric Mann-Whitney *t* test. Quadriceps and diaphragm mRNA content markers at 4 weeks all failed normality (MURF1, atrogin, UBC, Gadd45a) and were assessed by a nonparametric Mann-Whitney *t* test. All other data passed normality. A 2-tailed unpaired *t* test was used to compare C26 with PBS within each time point with respect to muscle mass, fiber CSA, remaining Western blots, quantitative PCR, and glutathione measures. A 2-way ANOVA with factors of time point (2 versus 4 week) and treatment (C26 versus PBS) was used for mitochondrial respiration, mH_2_O_2_, and all force-frequency experiments followed by Benjamini, Krieger, and Yekutieli’s post hoc analysis. (49) when a significant interaction was observed. All statistical analyses were performed with GraphPad Prism Software 8.4.2.

### Study approval

All experiments and procedures were approved by the Animal Care Committee at York University (AUP Approval Number 2019-10) in accordance with the Canadian Council on Animal Care.

## Author contributions

LJD, CAB, MERC, NPG, and CGRP contributed to the rationale and study design. LJD, CAB, SG, and CGRP conducted all experiments and/or analyzed all data. CGRP and LJD wrote the manuscript. LJD, CAB, SG, SND, MCG, AKT, ST, MERC, FAR, AJC, MPW, US, JQ, NPG, and CGRP contributed to the interpretation of the data and manuscript preparation. All authors have approved the final version of the manuscript and agree to be accountable for all aspects of the work. All persons designated as authors qualify for authorship, and all those who qualify for authorship are listed.

## Supplementary Material

Supplemental data

## Version 1 11/08/2022

In-Press Preview

## Version 2 12/22/2022

Electronic publication
