# Supplementary material for: Muscle weakness precedes atrophy during cancer cachexia and is linked to muscle-specific mitochondrial stress
Source: JCI Insight. 2022 Dec 22;7(24):e155147. doi: 10.1172/jci.insight.155147 (PMC9869968; doi:10.1172/jci.insight.155147)
Supplement: Supplemental data [file jciinsight-7-155147-s084.pdf]

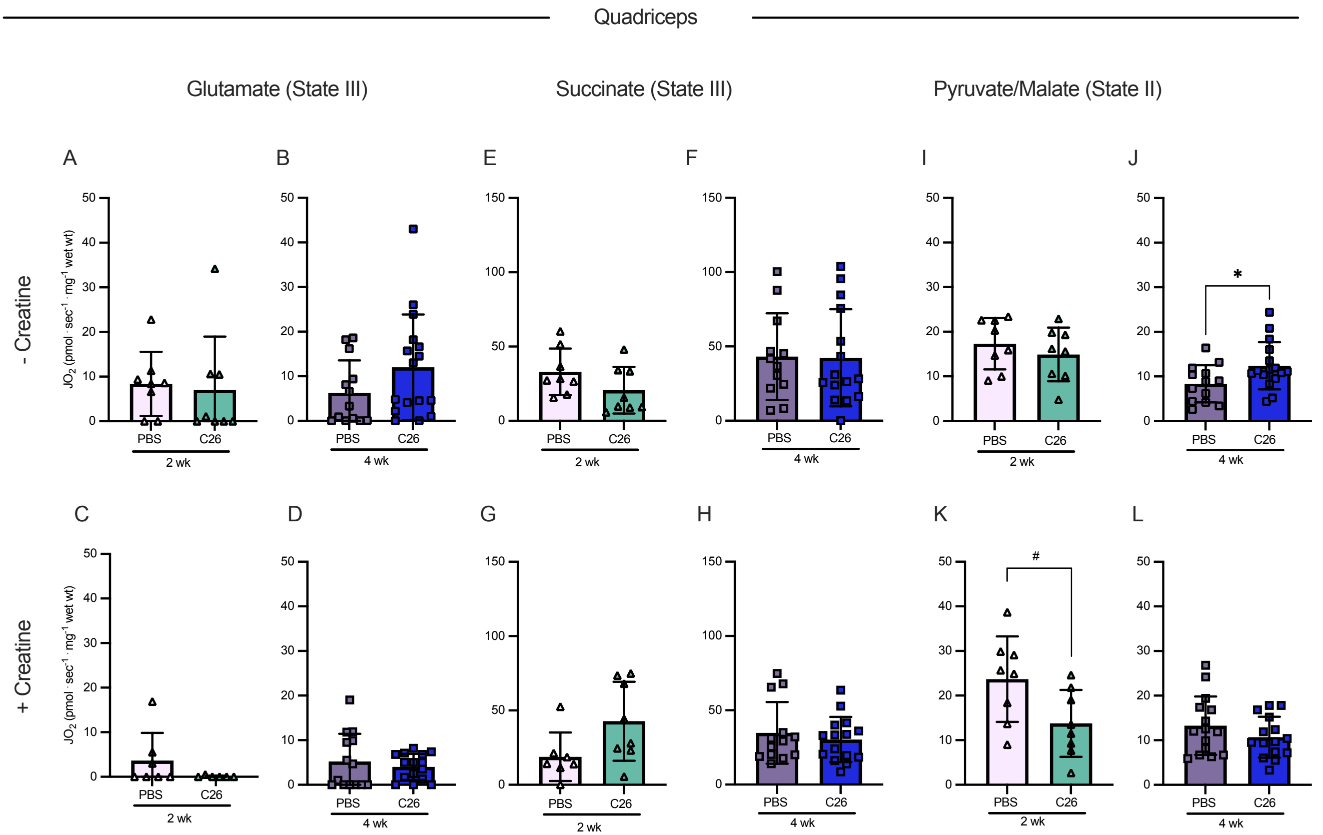

Quadriceps
Glutamate (State III)
Succinate (State III)
Pyruvate/Malate (State II)
- Creatine
+ Creatine
A
B
C
D
E
F
G
H
I
J
K
L
JO2 (pmol · sec-1 · mg-1 wet wt)
PBS
C26
2 wk
4 wk
*
#

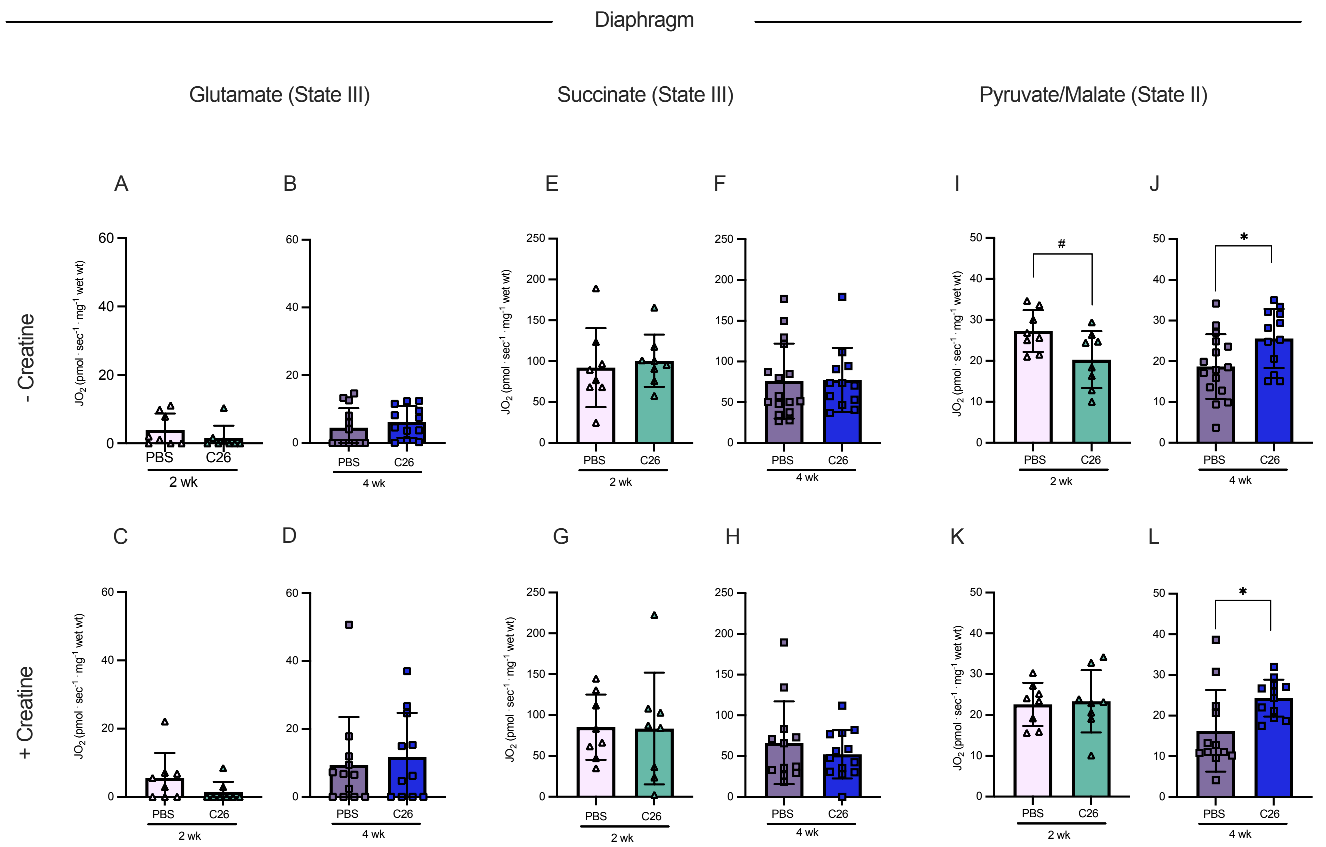

Diaphragm
Glutamate (State III)
Succinate (State III)
Pyruvate/Malate (State II)
- Creatine
+ Creatine
A
B
C
D
E
F
G
H
I
J
K
L
JO2 (pmol · sec-1 · mg-1 wet wt)
PBS
C26
2 wk
4 wk
#
*

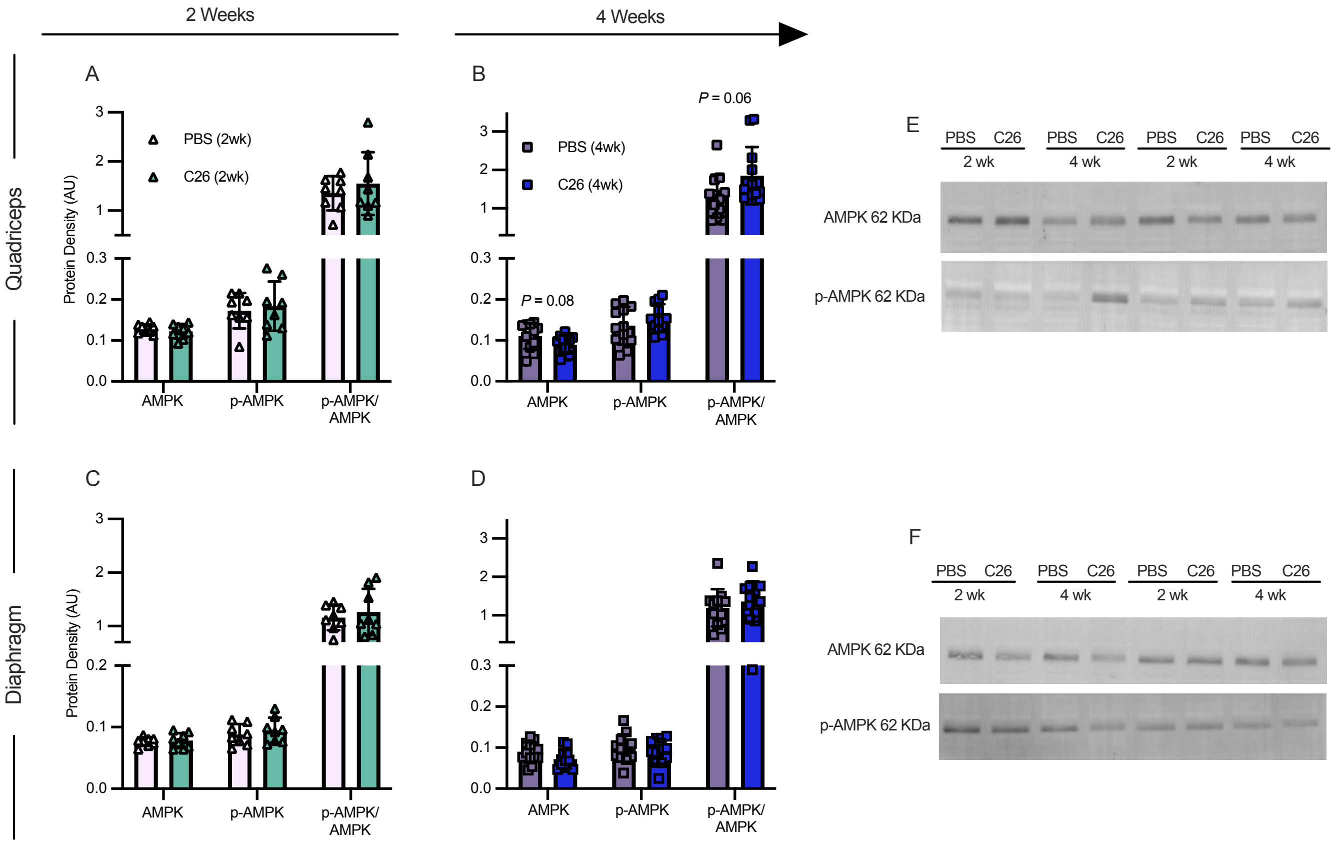

2 Weeks
4 Weeks
Quadriceps
Diaphragm
A
B
C
D
E
F
Protein Density (AU)
PBS (2wk)
C26 (2wk)
PBS (4wk)
C26 (4wk)
P = 0.06
P = 0.08
AMPK
p-AMPK
p-AMPK/ AMPK
PBS C26 PBS C26 PBS C26 PBS C26
2 wk 4 wk 2 wk 4 wk
AMPK 62 KDa
p-AMPK 62 KDa

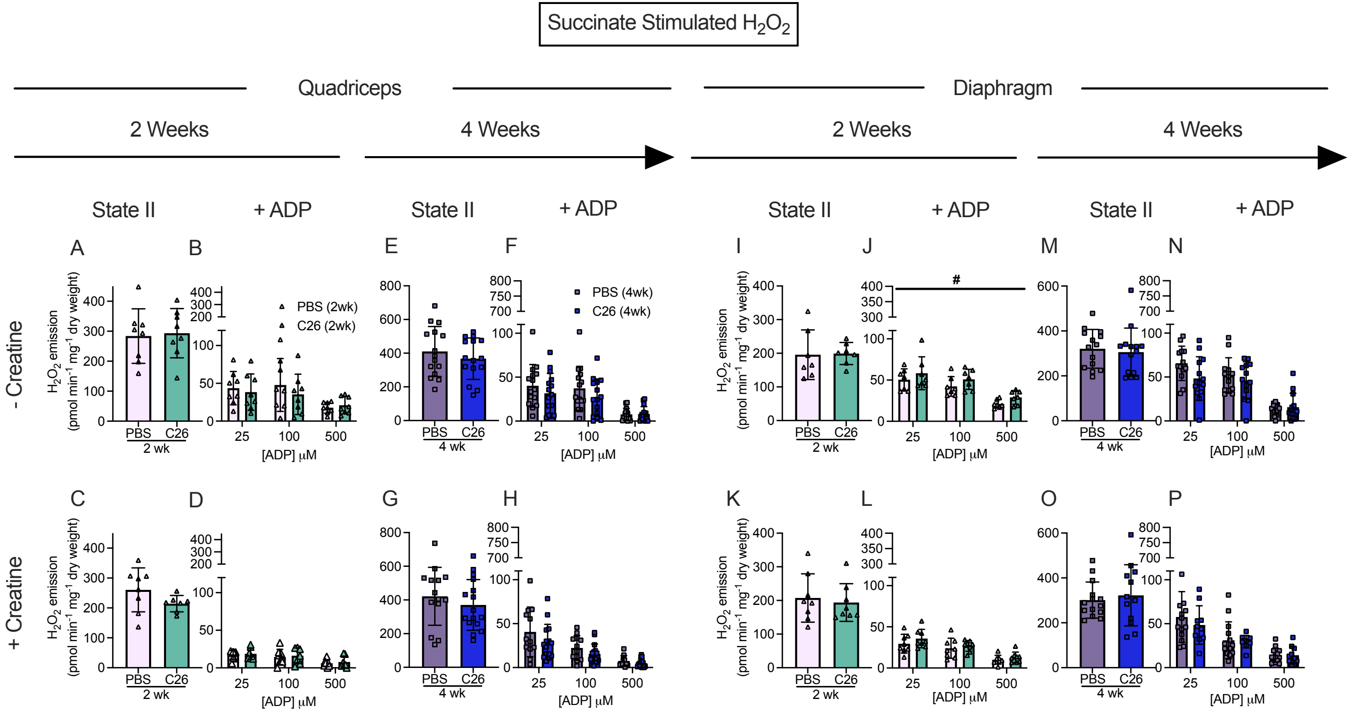

Succinate Stimulated H_2O_2
Quadriceps
Diaphragm
2 Weeks
4 Weeks
2 Weeks
4 Weeks
State II
+ ADP
- Creatine
+ Creatine
A
B
C
D
E
F
G
H
I
J
K
L
M
N
O
P
H_2O_2 emission (pmol min^{-1} mg^{-1} dry weight)
PBS
C26
2 wk
4 wk
[ADP] μM
PBS (2wk)
C26 (2wk)
PBS (4wk)
C26 (4wk)
#

Supplemental Figure Legends

Figure S1 Multiple substrate evaluation of oxygen consumption in quadriceps permeabilized muscle fibre bundles. Oxygen consumption was evaluated in the absence of creatine at 2 weeks and 4 weeks post C26 implantation or PBS injections in permeabilized muscle fibres when stimulated with glutamate (*A*, *B*), succinate (*E*, *F*) and pyruvate/malate (*I*, *J*). This was repeated in the presence of 20mM Creatine (*C*, *D*, *G*, *H*, *K*, *L*). Results represent mean ± SD; n=8-16; # $P<0.05$ PBS (2wk) vs C26 (2wk); * $P<0.05$ PBS (4wk) vs C26 (4wk)

Figure S2 Multiple substrate evaluation of oxygen consumption in diaphragm permeabilized muscle fibre bundles. Oxygen consumption was evaluated in the absence of creatine at 2 weeks and 4 weeks post C26 implantation or PBS injections in permeabilized muscle fibres when stimulated with glutamate (*A*, *B*), succinate (*E*, *F*) and pyruvate/malate (*I*, *J*). This was repeated in the presence of 20mM Creatine (*C*, *D*, *G*, *H*, *K*, *L*). Results represent mean ± SD; n=8-16; # $P<0.05$ PBS (2wk) vs C26 (2wk); * $P<0.05$ PBS (4wk) vs C26 (4wk).

Figure S3 Muscle-specific changes in markers of growth in C26 tumour-bearing skeletal muscle. Protein content of AMPKα and P-AMPKα were quantified at in the quadriceps at 2 weeks (*A*, *n*=8) and 4 weeks (*B*, *n*=12). Markers were also quantified at in the diaphragm at 2 weeks (*C*, *n*=8) and 4 weeks (*D*, *n*=12). *E*, representative image for quadriceps and *F*, representative image for diaphragm. Results represent mean ± SD.

Figure S4 Succinate stimulated mH_2O_2 emission in quadriceps and diaphragm muscle of C26 tumour bearing mice. At 2 and 4 weeks, quadriceps mH_2O_2 emission supported by succinate

(10mM) ($FADH_2$) was assessed under maximal State II (no ADP) conditions in the absence of creatine (*A*, *E*) and in the presence of 20mM Creatine (*C*, *G*). State III (range of [ADP] to model metabolic demand) was also assessed in the absence of creatine (*B*, *F*) and in the presence of 20mM creatine (*D* and *H*). These measures were repeated in the diaphragm (I-P). Results represent mean ± SD; n=7-16; # P<0.05, PBS (2wk) vs C26 (2wk); * P<0.05, PBS (4wk) vs C26 (4wk).
